# Mediation effects of metabolites and sex hormones on the relationship between body mass index and breast cancer: Mendelian randomization analysis and mediation analysis

**DOI:** 10.3389/fonc.2024.1449956

**Published:** 2024-11-21

**Authors:** Yanjiang Yang, Min Chen, Wenwen Yang

**Affiliations:** ^1^ Department of Rheumatology and Immunology, The People's Hospital of Qiandongnan Autonomous Prefecture, Kaili, Guizhou, China; ^2^ The First Clinical Medical College, Lanzhou University, Lanzhou, Gansu, China

**Keywords:** breast cancer, body mass index, sex hormones, metabolites, Mendelian randomization

## Abstract

**Background:**

Observational investigations have indicated a notable correlation between body mass index (BMI) and breast cancer (BC). Nevertheless, the precise biological pathways driving this correlation remain ambiguous. Consequently, we utilized Mendelian randomization (MR) techniques to explore the causative link between BMI and genetic predisposition to BC, as well as the potential intermediary influences.

**Methods:**

Utilizing extensive cohorts sourced from publicly accessible genome-wide association studies (GWAS) datasets of European populations, we conducted Mendelian randomization (MR) analysis. The primary method employed was the Inverse Variance Weighted (IVW) model. We evaluated both heterogeneity and horizontal pleiotropy. Our MR analysis unveiled several metabolites and sex hormones as mediators in the association between BMI and BC.

**Results:**

The IVW model indicated significant negative causal correlations between BMI and BC, ER^+^BC, and ER^-^BC. Thirty-five metabolites, thirty-three metabolites and sex hormones, and fifteen metabolites respectively mediated the causal effects of BMI on BC, ER+BC, and ER^-^BC. Furthermore, our study found that BMI influences BC risk through different mediating factors; BMI increases ER+BC risk through the pathway of sex hormones (biologically available testosterone) and decreases the causal relationship of BC risk through multiple metabolite pathways.

**Conclusion:**

This study discovered that BMI increases ER^+^BC risk through the pathway of sex hormones (biologically available testosterone), and decreases BC risk through multiple metabolite pathways causally. These discoveries could offer insights into the development of preventive strategies and interventions for BC, while further investigations should delve into alternative feasible biological pathways.

## 1 Introduction

Breast cancer (BC) ranks among the most prevalent malignant tumors in women, accounting for a considerable proportion of female malignancies (1). Globally, the incidence of BC is on the rise, despite advancements in early detection and treatment. Nonetheless, BC continues to stand as a prominent contributor to mortality among women attributed to cancer (2). The occurrence of BC involves various factors, including age, family history, reproductive factors, estrogen, and lifestyle (3). In recent years, the widespread utilization of genetic prediction causal inference methods has offered new insights into early screening and prevention strategies for complex diseases. This study aims to utilize genetic instruments to investigate body mass index (BMI) as a potential risk factor for BC and explore its potential mediating effects, offering fresh perspectives on early prevention and screening of BC.

BMI is closely associated with cancer occurrence. Research has indicated a specific association between BMI and the risk of BC, especially among those with obesity (including high BMI) (4). Nonetheless, the intricacy of this association is impacted by diverse elements, such as menopausal status and specific life stages. For postmenopausal individuals, some meta-analyses have highlighted a positive correlation between elevated obesity, adult weight gain, and the risk of hormone receptor-positive BC, including both estrogen receptor-positive (ER^+^) and progesterone receptor-positive subtypes (5–7). Conversely, epidemiological evidence suggests an inverse relationship or lack of association between high BMI and the risk of premenopausal hormone receptor-positive (8, 9). Studies (10, 11) have demonstrated that testosterone is converted into estrogen within adipocytes, and the upregulation of aromatase further increases the risk of breast cancer. In adipose tissue and normal breast tissue, aromatase is regulated by different promoters, activated by factors such as IL-6, IL-11, and TNF-α. In postmenopausal women, adipose tissue serves as the primary source of estrogen, and a positive correlation between circulating estradiol levels and BC risk has been confirmed (12, 13).

In recent years, modern “omics” technologies, including metabolomics, have significantly contributed to exploration of disease mechanisms. Metabolites, the end products or intermediates of metabolism, play critical roles in human physiology. By revealing altered metabolic pathways and intermediate metabolites, metabolomics provides novel insights into the biological mechanisms underlying diseases (14, 15).

Nevertheless, there is currently no Mendelian Randomization (MR) evidence to establish a causal relationship between BMI and BC, nor to elucidate the mechanisms by which BMI influences breast cancer risk. Building on prior research into sex hormones and metabolites about BC, we employed mediation analysis to investigate the mediating roles of sex hormones and metabolites in the BMI-BC relationship. Mediation analysis is a statistical approach aimed at identifying intermediate factors that may serve as potential intervention targets, thereby enhancing our understanding of the etiological components involved (16). Therefore, our objective is to investigate the causal connection between BMI and the overall susceptibility to BC, as well as the genetic predisposition to estrogen receptor-positive (ER^+^) and estrogen receptor-negative (ER^-^) BC. We hypothesize that the influence of BMI on BC risk factors might be mediated through metabolic or sex hormone pathways.

Given the abundance of information garnered from genome-wide association studies (GWAS) and the application of extensive sample sizes, the MR approach has become a valuable tool. MR utilizes genetic variations, frequently single nucleotide polymorphisms (SNPs), as instrumental variables (IVs) to evaluate causal links between exposures and disease outcomes. Furthermore, owing to the stochastic assortment and recombination of alleles during gametogenesis, genetic variation achieves a form of random allocation within the population, mirroring the principles of traditional randomized controlled trials (17). Consequently, the evidentiary standard of MR studies surpasses that of observational studies (18).

In this investigation, we conducted a two-step MR analysis to elucidate the intermediary pathways linking BMI to BC via metabolites and sex hormones. Building upon these premises, we conducted a two-sample MR and mediation analysis utilizing GWAS databases to comprehensively assess the genetic causal association between BMI and the risk of BC.

## 2 Materials and methods

### 2.1 Study design and MR hypotheses

**Figure 1:**
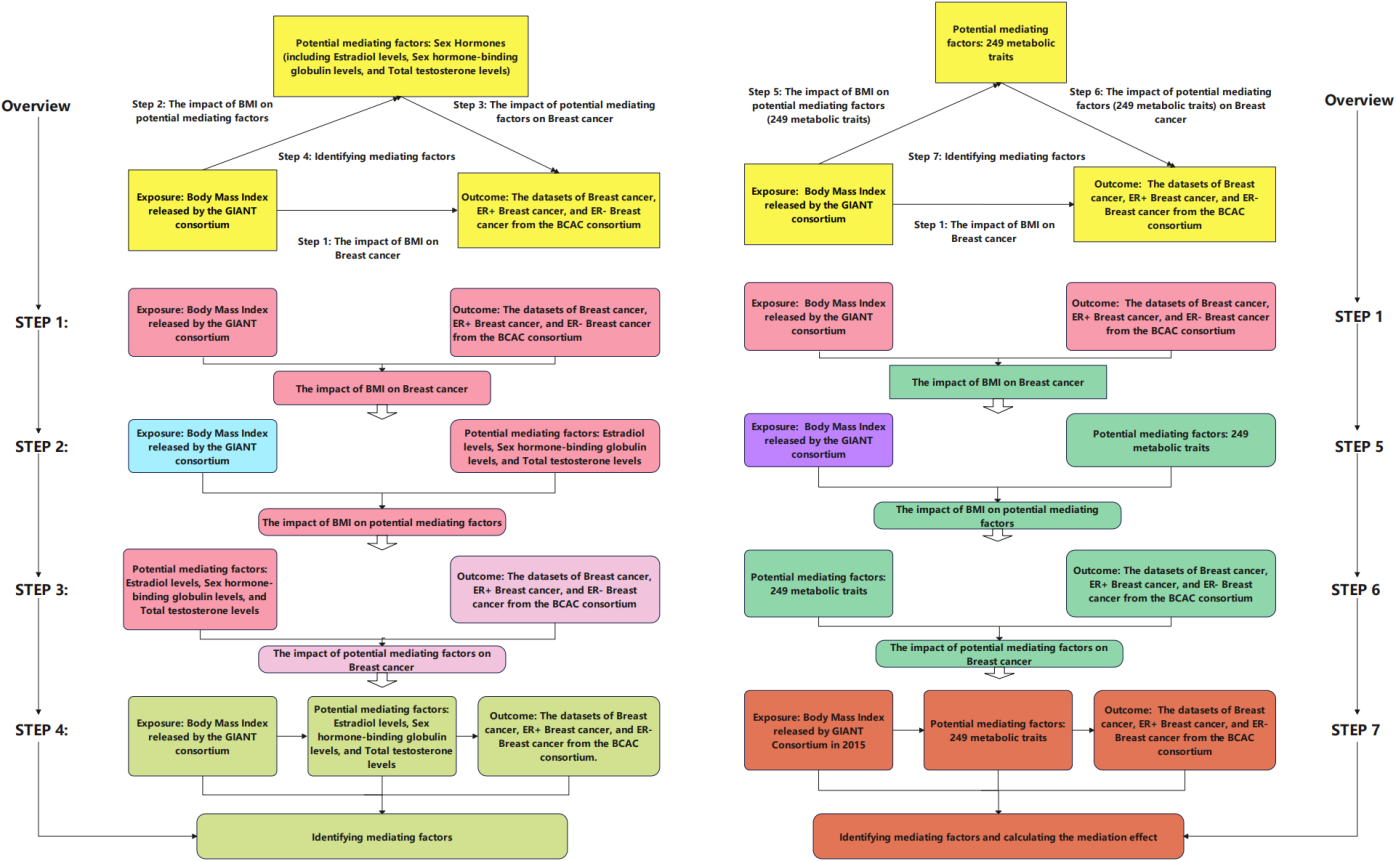
Flowchart of the study design. In this figure, we present our analytical process. In the left half of this figure, we outline four steps of our analysis: (1) examination of the impact of body mass index (BMI) on breast cancer (Step 1; corresponding table is **Table 2**); (2) analysis of the influence of BMI on hormones (Step 2; corresponding table is **Table 2**); (3) investigation of the effect of hormones on breast cancer (Step 3; corresponding table is **Table 2**); (4) determination of intermediate factors (Step 4; corresponding table is shown in **Table 2**). In the right half of this figure, we illustrate four steps of our analysis: (1) examination of the impact of BMI on breast cancer (Step 1; corresponding table is **Table 2**); (2) investigation of the influence of BMI on 249 metabolic features (Step 6; corresponding tables are **Supplementary Tables S1** and **S3**); (3) analysis of the impact of 249 metabolic features on breast cancer (Step 5; corresponding tables are **Supplementary Tables S2**, **S4**, and **S6**); (4) determination of intermediate factors, calculation of the ratio of mediation effect to total effect (Step 7; corresponding tables are shown in **Tables 3**–**5**, and **Supplementary Tables S3**, **S5**). Additionally, some analyses are not depicted in the figure: we selected metabolic features associated with breast cancer based on false discovery rate (FDR)-adjusted p-values (corresponding tables are **Supplementary Tables S7**-**S9**).

This study employed summary data sourced from the Bristol University (IEU) Open GWAS project, which encompasses GWAS summary data extracted from published literature, the UK Biobank, and the FinnGen Biobank. All datasets included in this study are anonymous, de-identified, and publicly available, thus not requiring approval from ethics review boards. To achieve credible causal inference, three assumptions must be satisfied for MR analysis IVs: (1) Instrumental variables necessitate a strong association with the exposure. (2) Instrumental variables must demonstrate independence from any potential confounding factors. (3) Instrumental variables must have no direct relationship with the outcome. **Figure 1** illustrates the roadmap of this study.

### 2.2 Data source

SNPs related to exposure factors (BMI) and mediator factors (sex hormones, 249 metabolites) were obtained from the IEU GWAS database project. The BMI stems from two datasets released by the Genetic Investigation of Anthropometric Traits (GIANT) consortium in 2015 and 2013, comprising sample sizes of 171,977 and 73,137, respectively. Notably, the dataset unveiled by the GIANT consortium in 2013 is employed as **Supplementary Data** in this context. Sex hormones in the mediator factors included Bioavailable testosterone levels, Estradiol levels, Sex hormone-binding globulin levels, and Total testosterone levels. GWAS data for BC were obtained from The Breast Cancer Association Consortium (BCAC), comprising datasets for BC, ER^+^BC, and ER^-^BC as outcome variables. Additional information is presented in **Table 1**.

**Table 1:** Information of the outcomes, mediating factors, and exposures.

		phenocode in IEU OpenGWAS project.	Sample size	The consortium, articles or biobank of the data source	Number of European-descent cases	Number of European-descent controls
exposure	Body mass index	ieu-a-974	171977	GIANT consortium released in 2015	NA	NA
	Body mass index	ieu-a-95	73137	GIANT consortium released in 2013	NA	NA
Mediating factors	Bioavailable testosterone levels	ebi-a-GCST90012102	188507	PMID:32042192	NA	NA
	Estradiol levels	ebi-a-GCST90020092	163985	PMID:34255042	37461	126524
	Sex hormone-binding globulin levels	ebi-a-GCST90012107	189473	PMID:32042192	NA	NA
	Total testosterone levels	ebi-a-GCST90012112	230454	PMID:32042192	NA	NA
	249 metabolic traits	ebi-a-GCST90092803 - ebi-a-GCST90093051	110051-115082	The UK Biobank	NA	NA
outcome	Breast cancer	ieu-a-1126	228951	BCAC consortium	122977	105974
	ER+ Breast cancer	ieu-a-1127	175475	BCAC consortium	69501	105974
	ER- Breast cancer	ieu-a-1128	127442	BCAC consortium	21468	105974

IEU, Integrative Epidemiology Unit; GWAS, Genome-Wide Association Studies; GIANT, the Genetic Investigation of ANthropometric Traits; BCAC, The Breast Cancer Association Consortium. More information about exposure, mediating factors,and outcomes is available at the IEU OpenGWAS project (https://gwas.mrcieu.ac.uk/).Estradiol was analyzed as a binary phenotype above/below detection limit (175 pmol/L).

### 2.3 Selection of IVs

Following the fundamental assumptions of the MR study, SNPs demonstrating correlations meeting the threshold of P < 5 × 10^−8^ were designated as instrumental variables following screening of the GWAS data. To mitigate the potential impact of linkage disequilibrium (LD) among SNPs on the analytical outcomes, stringent criteria were applied, including a parameter requirement of r^2^ < 0.001 and a fixed window size of 10,000 kb (19). To mitigate confounding effects, we excluded SNPs (P<10E-8) associated with the outcome during the analysis. Robust associations between instrumental and endogenous variables were ensured, while minimizing the risk of weak instrumental variable bias, through separate computations for each SNP. These computations encompassed the calculation of R^2^, indicating the proportion of variance explained by each instrumental variable SNP, alongside the utilization of the F statistic to assess the robustness of instrumental variables (20, 21).

### 2.4 Statistical analysis

We utilized the Inverse Variance Weighted (IVW) method as the primary means to assess causal effects, recognizing its robustness in identifying causal relationships within two-sample MR analysis (22). Our investigation involved a comparative analysis of outcomes obtained from the IVW method alongside those from the Weighted Median and MR-Egger methods. While the Weighted Median method accommodates up to 50% invalid instrumental variables (IVs), the MR-Egger method allows for the possibility of all IVs being invalid. Hence, when the three models are consistent, it is more convincing. We assessed the heterogeneity of the IVW model using Cochran’s Q test, wherein a significance level below p < 0.05 indicates the presence of heterogeneity. It is important to note, however, that the existence of heterogeneity does not necessarily signify the inefficacy of the IVW model. The MR-Egger method, which permits a non-zero intercept, was employed to identify directional pleiotropy. Additionally, we conducted the leave-one-out analysis to evaluate the impact of excluding individual SNPs on the results. The MR-PRESSO approach serves a dual purpose: it identifies outliers and offers a mechanism for assessing causality. Through this approach, it is possible to observe whether the presence of outliers affects the significance of the results. The IVW method, known for its heightened sensitivity in detecting causality, serves as our primary tool to ascertain the presence of a causal relationship. The False Discovery Rate (FDR) is a widely utilized correction method employed in multiple hypothesis testing. It serves to regulate the proportion of false positives within the pool of significant findings, thus enabling a more permissive analytical approach. All analyses were performed utilizing the TwoSampleMR package (23) in R software. Statistical significance in the analysis results was denoted by q<0.05, with q representing the adjusted p-value.

### 2.5 Mediation analysis

We conducted a two-step MR analysis to investigate mediation. Firstly, we computed the causal effect of BMI on the mediator (β1) and subsequently estimated the causal effect of the mediator on BC (β2). Z-values falling between -1.96 and 1.96, as per the normal distribution table, are commonly interpreted as within the 95% confidence interval, indicating statistically nonsignificant results. Consequently, the mediation effect was estimated based on β1×β2, with the sign of β1×β2 reflected by the Z-value. The significance of the mediation effect was assessed using the Delta method. This study examined the mediating influence of sex hormones and 249 metabolites on the causal association between BMI and BC. Specifically, the study investigated the role of sex hormones and 249 metabolites as mediators in the causal relationship between BMI and BC.

## 3 Results

### 3.1 Body mass index and its association with breast cancer

**Table 2** describes the MR results from several approaches to analyzing the causal effect of BMI on BC. The IVW results indicate significant negative causal relationships between BMI and BC, ER^+^ BC, and ER^-^ BC in two-sample MR analyses. The effect estimates for BMI on BC, ER^+^ BC, and ER^-^ BC were -0.184, -0.183, and -0.349, respectively, with corresponding q-values of 3.32E-03, 4.28E-03, and 1.03E-03.

**Table 2:** The mediators were screened according to their P-value (p<0.05) after the FDR corrected.

	outcome	exposure	Number of SNPs	IVW_Beta	IVW_se	IVW_pval	IVW_q_value	IVW_Bonferroni-adjusted p-value	Weighted_median_Beta	Weighted_median_se	Weighted_median_pval	MR_Egger_Beta	MR_Egger_se	MR_Egger_pval	Q	Q_pval	egger_intercept	se	pval	Raw_Causal. Estimate	Raw_Sd	Raw_P_value	Outlier-corrected_Causal. Estimate	Outlier-corrected_Sd	Outlier-corrected_P_value
The impact of female body mass index on Breast cancer	Breast cancer	Body mass index || id:ieu-a-974	34	-1.84E-01	6.02E-02	2.21E-03	3.32E-03	6.63E-03	-1.61E-01	6.32E-02	1.06E-02	-4.13E-01	1.84E-01	3.22E-02	8.95E+01	4.12E-07	8.17E-03	6.23E-03	1.99E-01	-1.84E-01	6.02E-02	4.37E-03	-2.02E-01	5.77E-02	1.38E-03
	ER+ Breast cancer	Body mass index || id:ieu-a-974	35	-1.83E-01	6.40E-02	4.28E-03	4.28E-03	1.28E-02	-1.68E-01	6.56E-02	1.05E-02	-2.66E-01	2.04E-01	2.01E-01	7.64E+01	4.21E-05	2.98E-03	6.93E-03	6.70E-01	-1.83E-01	6.40E-02	7.25E-03	-1.80E-01	5.78E-02	3.86E-03
	ER- Breast cancer	Body mass index || id:ieu-a-974	35	-3.49E-01	9.76E-02	3.43E-04	1.03E-03	1.03E-03	-3.70E-01	1.03E-01	3.07E-04	-6.02E-01	3.09E-01	6.00E-02	7.61E+01	4.57E-05	9.01E-03	1.05E-02	3.95E-01	-3.49E-01	9.76E-02	1.06E-03	-3.46E-01	8.97E-02	5.16E-04
The impact of female body mass index (GIANT consortium ID:ieu-a-974/ID:ieu-a-95) on sex hormones	Bioavailable testosterone levels || id:ebi-a-GCST90012102	Body mass index || id:ieu-a-974	37	1.23E-01	1.61E-02	2.05E-14	8.20E-14	8.20E-14	1.00E-01	2.15E-02	3.09E-06	4.25E-02	4.19E-02	3.17E-01	5.65E+01	1.59E-02	3.18E-03	1.53E-03	4.49E-02	1.23E-01	1.61E-02	4.73E-09	NA	NA	NA
	Estradiol levels || id:ebi-a-GCST90020092	Body mass index || id:ieu-a-974	29	-7.35E-02	7.16E-02	3.04E-01	3.46E-01	1	-4.01E-02	1.01E-01	6.92E-01	4.88E-02	1.90E-01	8.00E-01	3.24E+01	2.57E-01	-5.07E-03	7.30E-03	4.93E-01	-7.35E-02	7.16E-02	3.13E-01	NA	NA	NA
	Sex hormone-binding globulin levels || id:ebi-a-GCST90012107	Body mass index || id:ieu-a-974	34	-8.54E-02	1.36E-02	2.94E-10	5.88E-10	1.18E-09	-9.81E-02	1.35E-02	4.30E-13	-8.02E-02	4.38E-02	7.68E-02	8.95E+01	4.07E-07	-1.86E-04	1.47E-03	9.00E-01	-8.54E-02	1.36E-02	3.98E-07	-9.11E-02	1.19E-02	1.07E-08
	Total testosterone levels || id:ebi-a-GCST90012112	Body mass index || id:ieu-a-974	37	2.33E-02	2.48E-02	3.46E-01	3.46E-01	1	-3.12E-03	2.36E-02	8.95E-01	-8.01E-02	6.54E-02	2.29E-01	1.09E+02	3.29E-09	4.07E-03	2.39E-03	9.78E-02	2.33E-02	2.48E-02	3.52E-01	2.49E-02	1.97E-02	2.14E-01
	Bioavailable testosterone levels || id:ebi-a-GCST90012102	Body mass index || id:ieu-a-95	11	1.18E-01	1.93E-02	9.41E-10	NA	NA	1.12E-01	2.40E-02	3.00E-06	3.82E-02	7.56E-02	6.25E-01	1.32E+01	2.13E-01	4.26E-03	3.89E-03	3.02E-01	1.18E-01	1.93E-02	1.13E-04	NA	NA	NA
The impact of sex hormones on Breast cancer	Breast cancer	Bioavailable testosterone levels || id:ebi-a-GCST90012102	124	1.45E-01	4.55E-02	1.40E-03	2.80E-03	5.61E-03	2.97E-02	4.62E-02	5.21E-01	1.92E-01	9.07E-02	3.64E-02	3.79E+02	8.11E-28	-1.47E-03	2.48E-03	5.54E-01	1.45E-01	4.55E-02	1.78E-03	1.59E-01	3.99E-02	1.15E-04
	Breast cancer	Estradiol levels || id:ebi-a-GCST90020092	2	3.46E-01	1.96E-01	7.75E-02	1.03E-01	3.10E-01	NA	NA	NA	NA	NA	NA	1.01E+01	1.52E-03	NA	NA	NA	NA	NA	NA	NA	NA	NA
	Breast cancer	Sex hormone-binding globulin levels || id:ebi-a-GCST90012107	196	-7.60E-02	5.41E-02	1.60E-01	1.60E-01	6.40E-01	-1.68E-02	6.01E-02	7.79E-01	8.11E-03	9.51E-02	9.32E-01	5.45E+02	6.76E-35	-1.75E-03	1.62E-03	2.84E-01	-7.60E-02	5.41E-02	1.62E-01	-6.00E-02	4.99E-02	2.30E-01
	Breast cancer	Total testosterone levels || id:ebi-a-GCST90012112	196	1.25E-01	2.56E-02	9.82E-07	3.93E-06	3.93E-06	1.18E-01	3.16E-02	1.91E-04	1.90E-01	5.24E-02	3.78E-04	4.05E+02	7.80E-17	-2.35E-03	1.67E-03	1.61E-01	1.25E-01	2.56E-02	2.05E-06	1.30E-01	2.36E-02	1.14E-07
The impact of sex hormones on ER+ breast cancer	ER+ Breast cancer	Bioavailable testosterone levels || id:ebi-a-GCST90012102	126	2.01E-01	5.28E-02	1.35E-04	2.70E-04	5.41E-04	1.52E-01	5.70E-02	7.56E-03	2.45E-01	1.05E-01	2.15E-02	3.66E+02	1.83E-25	-1.36E-03	2.85E-03	6.34E-01	2.01E-01	5.28E-02	2.12E-04	2.15E-01	4.51E-02	5.11E-06
	ER+ Breast cancer	Estradiol levels || id:ebi-a-GCST90020092	2	3.99E-01	1.30E-01	2.13E-03	2.84E-03	8.51E-03	NA	NA	NA	NA	NA	NA	3.08E+00	7.92E-02	NA	NA	NA	NA	NA	NA	NA	NA	NA
	ER+ Breast cancer	Sex hormone-binding globulin levels || id:ebi-a-GCST90012107	197	-1.06E-01	6.10E-02	8.36E-02	8.36E-02	3.34E-01	-2.97E-02	6.84E-02	6.64E-01	-2.76E-02	1.07E-01	7.97E-01	4.92E+02	1.85E-27	-1.62E-03	1.83E-03	3.77E-01	-1.06E-01	6.10E-02	8.52E-02	-1.05E-01	5.68E-02	6.50E-02
	ER+ Breast cancer	Total testosterone levels || id:ebi-a-GCST90012112	196	1.72E-01	2.89E-02	2.70E-09	1.08E-08	1.08E-08	1.61E-01	3.73E-02	1.53E-05	2.59E-01	5.90E-02	1.81E-05	3.63E+02	3.11E-12	-3.19E-03	1.88E-03	9.07E-02	1.72E-01	2.89E-02	1.23E-08	1.83E-01	2.78E-02	3.71E-10
The impact of sex hormones on ER- breast cancer	ER- Breast cancer	Bioavailable testosterone levels || id:ebi-a-GCST90012102	126	-3.60E-02	6.89E-02	6.02E-01	6.02E-01	1	1.20E-02	8.72E-02	8.90E-01	1.45E-01	1.36E-01	2.90E-01	2.66E+02	2.89E-12	-5.66E-03	3.69E-03	1.27E-01	-3.60E-02	6.89E-02	6.03E-01	-2.42E-02	5.89E-02	6.82E-01
	ER- Breast cancer	Estradiol levels || id:ebi-a-GCST90020092	2	2.52E-01	4.16E-01	5.44E-01	6.02E-01	1	NA	NA	NA	NA	NA	NA	1.38E+01	2.03E-04	NA	NA	NA	NA	NA	NA	NA	NA	NA
	ER- Breast cancer	Sex hormone-binding globulin levels || id:ebi-a-GCST90012107	198	8.64E-02	7.79E-02	2.68E-01	6.02E-01	1	2.45E-01	1.06E-01	2.08E-02	1.62E-01	1.38E-01	2.40E-01	3.46E+02	2.75E-10	-1.56E-03	2.34E-03	5.05E-01	8.64E-02	7.79E-02	2.69E-01	7.43E-02	7.57E-02	3.28E-01
	ER- Breast cancer	Total testosterone levels || id:ebi-a-GCST90012112	198	-2.03E-02	3.63E-02	5.75E-01	6.02E-01	1	8.75E-03	5.55E-02	8.75E-01	1.90E-02	7.47E-02	8.00E-01	2.49E+02	6.79E-03	-1.42E-03	2.36E-03	5.47E-01	-2.03E-02	3.63E-02	5.76E-01	-2.56E-02	3.53E-02	4.70E-01

We initially analyzed the impact of female body mass index (BMI) on breast cancer. Considering sex hormones as potential mediators, we subsequently examined the effects of BMI on sex hormones and their subsequent influence on breast cancer. It is noteworthy that during our analysis of the effect of BMI (id: ieu-a-974) on bioavailable testosterone levels, horizontal pleiotropy was detected. Therefore, we utilized BMI from different years (id: ieu-a-95) as an alternative. Through our analysis, we have reached several conclusions: 1. Higher BMI decreases the risk of breast cancer, regardless of ER+ or ER- status. 2. Increasing BMI is associated with elevated bioavailable testosterone levels and decreased sex hormone-binding globulin (SHBG) levels, while it has no effect on estradiol levels and total testosterone levels. 3. Higher levels of estrogen and testosterone increase the risk of ER+ breast cancer, but are not associated with the risk of ER- breast cancer. 4. Elevated BMI influences ER+ breast cancer risk by reducing SHBG levels and thereby increasing bioavailable testosterone levels; 5.We discovered a pathway through which body mass index influences ER+ breast cancer, yet this pathway cannot account for why higher body mass index reduces breast cancer risk. The IVW method, known for its heightened sensitivity in detecting causality, serves as our primary tool to ascertain the presence of a causal relationship. FDR (False Discovery Rate) and Bonferroni-adjusted p-values are commonly used correction methods in multiple hypothesis testing. FDR controls the proportion of false positives among all significant results, allowing for a more liberal approach compared to Bonferroni correction, which maintains a stringent familywise error rate. Bonferroni correction divides the significance level by the number of comparisons, ensuring a conservative control over Type I errors. Both methods serve to adjust p-values to maintain appropriate statistical significance thresholds in the context of multiple comparisons The MR-Egger method is also employed for detecting horizontal pleiotropy due to its allowance for the presence of non-zero intercepts (The columns in the table corresponding to “egger_intercept”, “se”, and “pval”), a pval below 0.05 suggests the existence of horizontal pleiotropy. Heterogeneity was assessed using Cochran’s Q test (The columns in the table corresponding to “Q” and “Q_pval”). To evaluate the impact of outliers on result significance, we applied the MR-PRESSO method (The columns in the table corresponding to “Raw_Causal.Estimate”, “Raw_Sd”, “Raw_P_value”, “Outlier-corrected_Causal.Estimate”, “Outlier-corrected_Sd”, and “Outlier-corrected_P_value”; Raw represents the outcomes of the analysis encompassing all SNPs, while Outlier-corrected signifies the results excluding outliers. IVW: Inverse Variance Weighted method; Weighted_median: Weighted median method; MR_Egger: MR-Egger method; SNPs: Single-nucleotide polymorphisms; q_value: The P value post FDR method (Benjaminiand Hochberg) corrected; NA: not applicable; ER: Estrogen Receptor.

### 3.2 Exploring the influence of sex hormones on BC development and the mediating mechanisms

We initially analyzed the impact of female BMI on BC. Considering sex hormones as potential mediators, we subsequently examined the effects of BMI on sex hormones and their subsequent influence on BC. We used a two-step MR analysis for mediated MR analysis. In the initial stage, we computed the causal effect of BMI on the mediator (β1), while in the subsequent step, we determined the causal effect of the mediator on BC (β2). Ultimately, the mediation effect can be estimated based on β1×β2, with the sign of β1×β2 reflected by the Z-value.

#### 3.2.1 Impacts of sex hormones on the development of BC

As shown in **Table 2**, in the two-sample MR analysis of sex hormones and BC, IVW results show a significant positive causal relationship between Bioavailable testosterone, Total testosterone levels, and BC (effect estimates are 0.145 and 0.125, respectively); in the analysis of ER+ BC, Bioavailable testosterone, Total testosterone levels, and Estradiol levels show a positive causal relationship with ER+ BC (with effect estimates of 0.201, 0.399, and 0.172, respectively). However, in the two-sample MR analysis investigating the association between sex hormones and ER^-^BC, no statistically significant causal relationship between the two variables was observed.

#### 3.2.2 Sex hormones act as mediators in the relationship between BMI increase and BC risk

A two-step methodology was utilized to conduct mediation MR analysis. Subsequent to completing the causal MR analyses assessing the influence of BMI on BC and sex hormones on BC, an investigation was initiated to examine whether the causal link between BMI and BC could be mediated through sex hormones (e.g., Bioavailable testosterone levels). It is noteworthy that during our analysis of the effect of BMI (using the dataset released by the GIANT consortium in 2015) on bioavailable testosterone levels, horizontal pleiotropy was detected. Therefore, we utilized BMI data from different years (using the dataset released by the GIANT consortium in 2013) as an alternative. The results, as presented in **Table 2**, indicate that BMI increases the risk of ER^+^BC through bioavailable testosterone, with a mediation effect estimate (β1×β2) of 0.025.

From **Table 2**, it can be observed that increasing BMI is associated with increased levels of bioavailable testosterone and decreased levels of sex hormone-binding globulin (SHBG), while it does not affect estradiol levels and total testosterone levels. Additionally, elevated levels of estrogen and testosterone are correlated with an increased risk of ER+ BC, while showing no association with the risk of ER-BC

### 3.3 Analyzing metabolic traits as mediators of the causal link between BMI and BC

In the preliminary analysis, the 249 metabolites were classified into nine main categories, comprising amino acids, low molecular weight metabolites, phospholipids, triglycerides, total lipids, (un)saturated fatty acids, cholesterol esters, free cholesterol, and lipoproteins. Among these metabolites, BMI exhibited a significant causal relationship with 183 metabolites (p < 0.05). **Supplementary Tables S2**, **S4**, and **S6** respectively present the MR results of 249 metabolites on BC, ER^+^ BC, and ER^-^ BC. **Supplementary Figure S1** illustrates the visual results of MR analysis of BMI on 249 metabolites and 249 metabolites on BC, ER^+^ BC, and ER^-^ BC. The final analysis results consistently indicate negative mediation effect estimates (β1×β2).

#### 3.3.1 Analysis of metabolite-mediated relationship between BMI and BC

**Figure 2:**
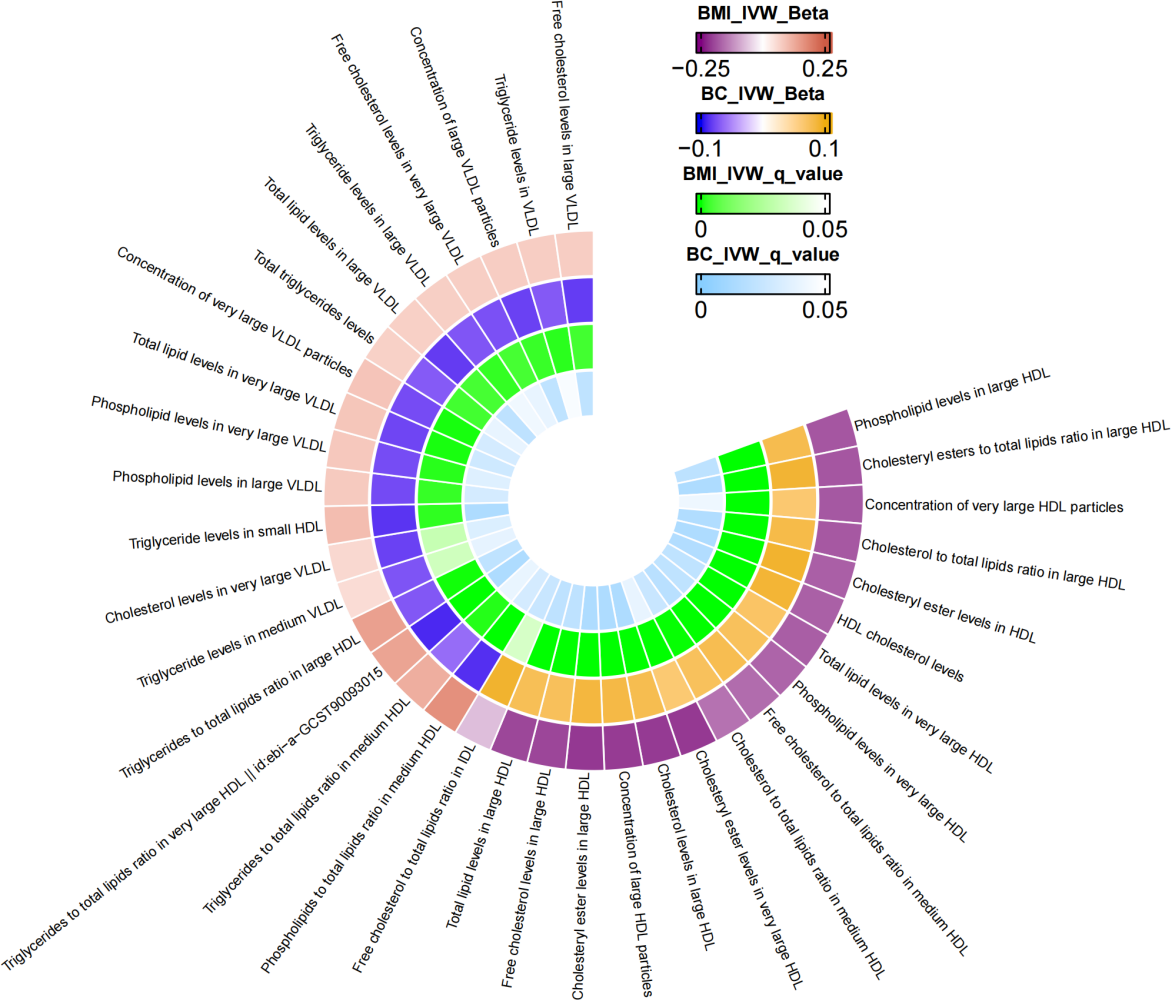
The mediator of 35 metabolic traits between BMI and BC causality. We visualized that 35 metabolic traits mediate the effect of BMI on breast cancer. The outermost boundary of the circular heat map consists of 35 metabolic traits and their corresponding identifiers. From the outermost to the innermost rings, the first and second rings of the circular heat map respectively depict the beta values of BMI on a particular metabolic trait and the beta values of the same trait on breast cancer (IVW method), while the third and fourth rings represent the q-values of these two aforementioned effects (IVW method).

**Supplementary Table S2** presents the results of the MR analysis investigating the influence of 249 metabolic traits on BC risk, identified by the BCAC consortium. The two-sample MR outcomes reveal that 79 metabolic traits demonstrate a causal effect on BC. Through screening potential metabolic traits affecting BC based on FDR-adjusted q-values (excluding those with horizontal pleiotropy), **Supplementary Table S3** highlights the selected candidates. After FDR correction, we identified 38 metabolic traits with q-values below 0.05 as mediator factors between BMI and BC, as depicted in **Supplementary Table S7**. Further data processing involved excluding mediators with horizontal pleiotropy and those with q-values below 0.05 in the relationship between BMI and BC. The final analysis results, presented in **Table 3**, unveil 35 metabolic traits mediating the negative causal association between BMI and BC. These traits predominantly encompass triglyceride levels (especially in VLDL), various cholesterol forms (notably HDL), concentrations of four lipoproteins, three phospholipids, and two total lipids. **Figure 2** provides a visual representation of the MR analysis, illustrating the mediation of 35 metabolic traits between BMI and BC causality.

**Table 3:** The mediators between body mass index and breast cancer were screened according to their P-value (p<0.05) after the FDR corrected (mediators with horizontal pleiotropy were excluded).

	The impact of body mass index (id:ieu-a-974) on potential mediators										The impact of potential mediators on breast cancer (id:ieu-a-1126)											
	outcome	exposure	Number of SNPs	IVW_Beta	IVW_se	IVW_pval	IVW_q_value	egger_intercept	se	pval	outcome	exposure	Number of SNPs	IVW_Beta	IVW_se	IVW_pval	IVW_q_value	egger_intercept	se	pval	Z	BMI_IVW_Beta * BC_IVW_Beta
1	HDL cholesterol levels || id:ebi-a-GCST90092822	Body mass index || id:ieu-a-974	34	-0.173062555	0.034780511	6.50E-07	3.30E-06	-0.003464685	0.003458168	0.32391551	Breast cancer (Combined Oncoarray; iCOGS; GWAS meta-analysis) || id:ieu-a-1126	HDL cholesterol levels || id:ebi-a-GCST90092822	72	0.089986833	0.026334123	0.000632876	0.015758611	0.003454631	0.002523162	0.175324133	-2.407426106	-0.015573351
2	Cholesteryl ester levels in HDL || id:ebi-a-GCST90092823	Body mass index || id:ieu-a-974	34	-0.173433271	0.03502089	7.33E-07	3.65E-06	-0.003309471	0.003487492	0.349755825	Breast cancer (Combined Oncoarray; iCOGS; GWAS meta-analysis) || id:ieu-a-1126	Cholesteryl ester levels in HDL || id:ebi-a-GCST90092823	76	0.092123476	0.025677245	0.000333547	0.014175255	0.003226356	0.002349855	0.173898093	-2.451195105	-0.015977276
3	Free cholesterol to total lipids ratio in IDL || id:ebi-a-GCST90092836	Body mass index || id:ieu-a-974	37	-0.069114589	0.030894472	0.025278611	0.035967853	-0.000135205	0.00310351	0.965498605	Breast cancer (Combined Oncoarray; iCOGS; GWAS meta-analysis) || id:ieu-a-1126	Free cholesterol to total lipids ratio in IDL || id:ebi-a-GCST90092836	49	0.092327361	0.030194726	0.002230194	0.025241739	-0.000856462	0.002726292	0.754798053	-1.81718134	-0.006381168
4	Cholesterol levels in large HDL || id:ebi-a-GCST90092844	Body mass index || id:ieu-a-974	33	-0.211846231	0.024722028	1.04E-17	8.65E-16	-0.003711157	0.002374461	0.128217192	Breast cancer (Combined Oncoarray; iCOGS; GWAS meta-analysis) || id:ieu-a-1126	Cholesterol levels in large HDL || id:ebi-a-GCST90092844	94	0.080101508	0.022514877	0.000374095	0.014175255	-7.52E-05	0.001966114	0.969589111	-3.063536846	-0.016969203
5	Cholesterol to total lipids ratio in large HDL || id:ebi-a-GCST90092845	Body mass index || id:ieu-a-974	35	-0.181553407	0.034643843	1.60E-07	1.29E-06	-0.002414184	0.00346177	0.490446756	Breast cancer (Combined Oncoarray; iCOGS; GWAS meta-analysis) || id:ieu-a-1126	Cholesterol to total lipids ratio in large HDL || id:ebi-a-GCST90092845	76	0.082598772	0.023931693	0.000557606	0.015427105	-0.000566253	0.002140483	0.792095935	-2.274541025	-0.014996088
6	Cholesteryl ester levels in large HDL || id:ebi-a-GCST90092846	Body mass index || id:ieu-a-974	33	-0.214121746	0.02510269	1.47E-17	9.12E-16	-0.003614961	0.002418553	0.145108841	Breast cancer (Combined Oncoarray; iCOGS; GWAS meta-analysis) || id:ieu-a-1126	Cholesteryl ester levels in large HDL || id:ebi-a-GCST90092846	95	0.086711546	0.022164889	9.15E-05	0.014175255	0.000494002	0.001911097	0.796598823	-3.252593095	-0.018566828
7	Cholesteryl esters to total lipids ratio in large HDL || id:ebi-a-GCST90092847	Body mass index || id:ieu-a-974	35	-0.183038567	0.033628091	5.24E-08	5.43E-07	-0.001958767	0.003367782	0.564775409	Breast cancer (Combined Oncoarray; iCOGS; GWAS meta-analysis) || id:ieu-a-1126	Cholesteryl esters to total lipids ratio in large HDL || id:ebi-a-GCST90092847	69	0.090484759	0.025552911	0.000398501	0.014175255	0.000170272	0.002266797	0.940346561	-2.518897182	-0.016562201
8	Free cholesterol levels in large HDL || id:ebi-a-GCST90092848	Body mass index || id:ieu-a-974	33	-0.200195714	0.023362377	1.04E-17	8.65E-16	-0.004020638	0.002216042	0.079315506	Breast cancer (Combined Oncoarray; iCOGS; GWAS meta-analysis) || id:ieu-a-1126	Free cholesterol levels in large HDL || id:ebi-a-GCST90092848	81	0.075478679	0.023287585	0.001190463	0.021038424	0.000976861	0.00214867	0.65061861	-3.024259188	-0.015110508
9	Total lipid levels in large HDL || id:ebi-a-GCST90092850	Body mass index || id:ieu-a-974	33	-0.199036508	0.024246616	2.23E-16	1.11E-14	-0.004149204	0.002301235	0.081111832	Breast cancer (Combined Oncoarray; iCOGS; GWAS meta-analysis) || id:ieu-a-1126	Total lipid levels in large HDL || id:ebi-a-GCST90092850	85	0.077615676	0.023954601	0.001194813	0.021038424	0.000753165	0.002152039	0.727242106	-2.987080024	-0.015448353
10	Concentration of large HDL particles || id:ebi-a-GCST90092851	Body mass index || id:ieu-a-974	33	-0.210683268	0.023819901	9.17E-19	2.28E-16	-0.0039512	0.002267858	0.091377589	Breast cancer (Combined Oncoarray; iCOGS; GWAS meta-analysis) || id:ieu-a-1126	Concentration of large HDL particles || id:ebi-a-GCST90092851	87	0.084069178	0.022696236	0.000212141	0.014175255	0.001933954	0.002022998	0.341791317	-3.29896723	-0.017711969
11	Phospholipid levels in large HDL || id:ebi-a-GCST90092852	Body mass index || id:ieu-a-974	33	-0.183567261	0.024583683	8.20E-14	2.04E-12	-0.004357706	0.002324307	0.070263509	Breast cancer (Combined Oncoarray; iCOGS; GWAS meta-analysis) || id:ieu-a-1126	Phospholipid levels in large HDL || id:ebi-a-GCST90092852	75	0.080317232	0.024365697	0.00097959	0.020326488	0.001454089	0.002311998	0.531356965	-2.997388535	-0.014743614
12	Triglycerides to total lipids ratio in large HDL || id:ebi-a-GCST90092855	Body mass index || id:ieu-a-974	37	0.145148989	0.03851432	0.000164099	0.00037146	0.004688692	0.003786039	0.223804034	Breast cancer (Combined Oncoarray; iCOGS; GWAS meta-analysis) || id:ieu-a-1126	Triglycerides to total lipids ratio in large HDL || id:ebi-a-GCST90092855	66	-0.071762248	0.022650628	0.001533737	0.021211842	-0.000743177	0.002341646	0.751993164	-1.789164664	-0.010416218
13	Free cholesterol levels in large VLDL || id:ebi-a-GCST90092872	Body mass index || id:ieu-a-974	37	0.076359311	0.02474134	0.002026611	0.003528855	0.003628415	0.002408516	0.140913601	Breast cancer (Combined Oncoarray; iCOGS; GWAS meta-analysis) || id:ieu-a-1126	Free cholesterol levels in large VLDL || id:ebi-a-GCST90092872	54	-0.083075094	0.026477841	0.001703762	0.021211842	-0.000578947	0.002783412	0.836042143	-2.187738748	-0.006343557
14	Total lipid levels in large VLDL || id:ebi-a-GCST90092874	Body mass index || id:ieu-a-974	37	0.071556431	0.023482238	0.002309396	0.003938627	0.002834272	0.002309797	0.227988122	Breast cancer (Combined Oncoarray; iCOGS; GWAS meta-analysis) || id:ieu-a-1126	Total lipid levels in large VLDL || id:ebi-a-GCST90092874	59	-0.083120468	0.025787648	0.001267375	0.021038424	-0.001719892	0.002500847	0.49441582	-2.183811041	-0.005947804
15	Concentration of large VLDL particles || id:ebi-a-GCST90092875	Body mass index || id:ieu-a-974	37	0.075302748	0.023574035	0.001401661	0.002566276	0.002964418	0.002314538	0.208692614	Breast cancer (Combined Oncoarray; iCOGS; GWAS meta-analysis) || id:ieu-a-1126	Concentration of large VLDL particles || id:ebi-a-GCST90092875	59	-0.080967749	0.025691002	0.001623788	0.021211842	-0.001488949	0.002581948	0.566428466	-2.229572991	-0.006097094
16	Phospholipid levels in large VLDL || id:ebi-a-GCST90092876	Body mass index || id:ieu-a-974	37	0.081157023	0.025262376	0.001315554	0.002426466	0.003620578	0.002462826	0.150470237	Breast cancer (Combined Oncoarray; iCOGS; GWAS meta-analysis) || id:ieu-a-1126	Phospholipid levels in large VLDL || id:ebi-a-GCST90092876	59	-0.077109375	0.025942177	0.002955242	0.030291847	8.49E-05	0.002629635	0.974365659	-2.184719116	-0.006257967
17	Triglyceride levels in large VLDL || id:ebi-a-GCST90092878	Body mass index || id:ieu-a-974	37	0.072680165	0.022316042	0.001126531	0.002125047	0.002089648	0.002213864	0.351695451	Breast cancer (Combined Oncoarray; iCOGS; GWAS meta-analysis) || id:ieu-a-1126	Triglyceride levels in large VLDL || id:ebi-a-GCST90092878	59	-0.072663206	0.026798839	0.006699345	0.042772744	-0.001386475	0.002582443	0.593436526	-2.083890239	-0.005281174
18	Cholesterol to total lipids ratio in medium HDL || id:ebi-a-GCST90092893	Body mass index || id:ieu-a-974	36	-0.152662441	0.03735168	4.37E-05	0.000112333	-0.004337578	0.00366462	0.244766747	Breast cancer (Combined Oncoarray; iCOGS; GWAS meta-analysis) || id:ieu-a-1126	Cholesterol to total lipids ratio in medium HDL || id:ebi-a-GCST90092893	78	0.074665358	0.0243014	0.002122941	0.025172013	-0.000430499	0.002369429	0.856310671	-1.904868837	-0.011398596
19	Free cholesterol to total lipids ratio in medium HDL || id:ebi-a-GCST90092897	Body mass index || id:ieu-a-974	34	-0.159565519	0.033954669	2.61E-06	1.02E-05	-0.003983125	0.003355604	0.243962328	Breast cancer (Combined Oncoarray; iCOGS; GWAS meta-analysis) || id:ieu-a-1126	Free cholesterol to total lipids ratio in medium HDL || id:ebi-a-GCST90092897	75	0.079582606	0.023806508	0.000829099	0.018767784	0.001820823	0.002355762	0.442063427	-2.212423743	-0.01269864
20	Phospholipids to total lipids ratio in medium HDL || id:ebi-a-GCST90092901	Body mass index || id:ieu-a-974	34	0.170200915	0.033523685	3.83E-07	2.39E-06	0.002192949	0.003363125	0.519020883	Breast cancer (Combined Oncoarray; iCOGS; GWAS meta-analysis) || id:ieu-a-1126	Phospholipids to total lipids ratio in medium HDL || id:ebi-a-GCST90092901	74	-0.087905434	0.029698334	0.00307694	0.030291847	0.001292609	0.002777317	0.643038082	-2.384450776	-0.014961585
21	Triglycerides to total lipids ratio in medium HDL || id:ebi-a-GCST90092903	Body mass index || id:ieu-a-974	37	0.123780062	0.035964667	0.000578043	0.001170185	0.004437306	0.003533479	0.217511109	Breast cancer (Combined Oncoarray; iCOGS; GWAS meta-analysis) || id:ieu-a-1126	Triglycerides to total lipids ratio in medium HDL || id:ebi-a-GCST90092903	67	-0.06254868	0.022707562	0.005877634	0.039554891	0.000536408	0.002494706	0.830426032	-1.65688294	-0.007742279
22	Triglyceride levels in medium VLDL || id:ebi-a-GCST90092926	Body mass index || id:ieu-a-974	37	0.052254431	0.023077134	0.023553336	0.033705636	0.001803148	0.002298134	0.437958634	Breast cancer (Combined Oncoarray; iCOGS; GWAS meta-analysis) || id:ieu-a-1126	Triglyceride levels in medium VLDL || id:ebi-a-GCST90092926	57	-0.073182341	0.026035984	0.004941616	0.038451948	-0.000100588	0.002577703	0.969013813	-1.695902425	-0.003824102
23	Triglyceride levels in small HDL || id:ebi-a-GCST90092954	Body mass index || id:ieu-a-974	37	0.100675652	0.030480432	0.0009567	0.001832449	0.004031473	0.002984989	0.185497634	Breast cancer (Combined Oncoarray; iCOGS; GWAS meta-analysis) || id:ieu-a-1126	Triglyceride levels in small HDL || id:ebi-a-GCST90092954	63	-0.086135602	0.022880029	0.000166774	0.014175255	-0.003255387	0.002534012	0.203762357	-2.377785534	-0.008671758
24	Total triglycerides levels || id:ebi-a-GCST90092992	Body mass index || id:ieu-a-974	37	0.070282804	0.022789316	0.002042209	0.00353132	0.001705823	0.002271135	0.457620132	Breast cancer (Combined Oncoarray; iCOGS; GWAS meta-analysis) || id:ieu-a-1126	Total triglycerides levels || id:ebi-a-GCST90092992	63	-0.07037283	0.025451909	0.005693444	0.039379656	0.00028055	0.002534128	0.912210683	-2.058412738	-0.004946
25	Triglyceride levels in VLDL || id:ebi-a-GCST90093003	Body mass index || id:ieu-a-974	37	0.075888196	0.022581284	0.000777543	0.001548865	0.002050699	0.002241845	0.366588931	Breast cancer (Combined Oncoarray; iCOGS; GWAS meta-analysis) || id:ieu-a-1126	Triglyceride levels in VLDL || id:ebi-a-GCST90093003	61	-0.07070719	0.0264355	0.007479482	0.046559775	-0.000275056	0.002557385	0.914714767	-2.116008774	-0.005365841
26	Cholesteryl ester levels in very large HDL || id:ebi-a-GCST90093006	Body mass index || id:ieu-a-974	34	-0.212849589	0.026591055	1.20E-15	4.98E-14	-0.004153975	0.002541393	0.111949438	Breast cancer (Combined Oncoarray; iCOGS; GWAS meta-analysis) || id:ieu-a-1126	Cholesteryl ester levels in very large HDL || id:ebi-a-GCST90093006	83	0.06508984	0.023358891	0.005327857	0.039379656	-0.001099121	0.00217102	0.614042589	-2.363998787	-0.013854346
27	Total lipid levels in very large HDL || id:ebi-a-GCST90093010	Body mass index || id:ieu-a-974	35	-0.175259513	0.033124116	1.22E-07	1.12E-06	-0.005452382	0.003196603	0.097464741	Breast cancer (Combined Oncoarray; iCOGS; GWAS meta-analysis) || id:ieu-a-1126	Total lipid levels in very large HDL || id:ebi-a-GCST90093010	72	0.070886625	0.022517345	0.001643412	0.021211842	-0.001627551	0.002001634	0.418911304	-2.063454924	-0.012423555
28	Concentration of very large HDL particles || id:ebi-a-GCST90093011	Body mass index || id:ieu-a-974	35	-0.181716257	0.033083986	3.96E-08	4.29E-07	-0.005254691	0.00320239	0.110323833	Breast cancer (Combined Oncoarray; iCOGS; GWAS meta-analysis) || id:ieu-a-1126	Concentration of very large HDL particles || id:ebi-a-GCST90093011	78	0.065737875	0.024128019	0.006439015	0.042192493	-0.001617791	0.00218883	0.462115396	-1.921258305	-0.011945641
29	Phospholipid levels in very large HDL || id:ebi-a-GCST90093012	Body mass index || id:ieu-a-974	35	-0.167812821	0.032473708	2.37E-07	1.71E-06	-0.005819119	0.003108208	0.070066462	Breast cancer (Combined Oncoarray; iCOGS; GWAS meta-analysis) || id:ieu-a-1126	Phospholipid levels in very large HDL || id:ebi-a-GCST90093012	68	0.074894153	0.023588237	0.001498036	0.021211842	-0.00120089	0.002126252	0.574129936	-2.193899187	-0.012568199
30	Triglycerides to total lipids ratio in very large HDL || id:ebi-a-GCST90093015	Body mass index || id:ieu-a-974	37	0.139560841	0.032175611	1.44E-05	4.49E-05	0.00441667	0.003144588	0.168973719	Breast cancer (Combined Oncoarray; iCOGS; GWAS meta-analysis) || id:ieu-a-1126	Triglycerides to total lipids ratio in very large HDL || id:ebi-a-GCST90093015	62	-0.090361443	0.024943574	0.000291618	0.014175255	-0.001608051	0.002797731	0.567595941	-2.509943929	-0.012610919
31	Cholesterol levels in very large VLDL || id:ebi-a-GCST90093016	Body mass index || id:ieu-a-974	37	0.058601199	0.025474268	0.02142514	0.031016627	0.004016278	0.002467298	0.112536591	Breast cancer (Combined Oncoarray; iCOGS; GWAS meta-analysis) || id:ieu-a-1126	Cholesterol levels in very large VLDL || id:ebi-a-GCST90093016	53	-0.080873792	0.02803732	0.003920294	0.033660453	7.78E-05	0.0029832	0.979298963	-1.745743467	-0.004739301
32	Free cholesterol levels in very large VLDL || id:ebi-a-GCST90093020	Body mass index || id:ieu-a-974	37	0.074265467	0.024352072	0.002291029	0.003934249	0.003398832	0.002377899	0.161771377	Breast cancer (Combined Oncoarray; iCOGS; GWAS meta-analysis) || id:ieu-a-1126	Free cholesterol levels in very large VLDL || id:ebi-a-GCST90093020	56	-0.074620138	0.026957129	0.005638346	0.039379656	-0.00090347	0.002677908	0.737139004	-2.048683961	-0.005541699
33	Total lipid levels in very large VLDL || id:ebi-a-GCST90093022	Body mass index || id:ieu-a-974	37	0.087178533	0.023983503	0.000278048	0.000602034	0.003320101	0.002343057	0.165324673	Breast cancer (Combined Oncoarray; iCOGS; GWAS meta-analysis) || id:ieu-a-1126	Total lipid levels in very large VLDL || id:ebi-a-GCST90093022	58	-0.079078346	0.026215466	0.002557308	0.027685639	-0.001410772	0.002677041	0.600282447	-2.341399696	-0.006893934
34	Concentration of very large VLDL particles || id:ebi-a-GCST90093023	Body mass index || id:ieu-a-974	37	0.089416778	0.024488553	0.000260845	0.00056974	0.003495901	0.002388017	0.152136127	Breast cancer (Combined Oncoarray; iCOGS; GWAS meta-analysis) || id:ieu-a-1126	Concentration of very large VLDL particles || id:ebi-a-GCST90093023	61	-0.076581019	0.026150494	0.003406312	0.030291847	-6.59E-05	0.002627538	0.980089354	-2.30764482	-0.006847628
35	Phospholipid levels in very large VLDL || id:ebi-a-GCST90093024	Body mass index || id:ieu-a-974	37	0.083345498	0.024602211	0.000704752	0.001415187	0.003432086	0.002402401	0.161981484	Breast cancer (Combined Oncoarray; iCOGS; GWAS meta-analysis) || id:ieu-a-1126	Phospholipid levels in very large VLDL || id:ebi-a-GCST90093024	58	-0.076505258	0.026712735	0.004183287	0.034721284	-0.00095702	0.002694108	0.723754149	-2.202542346	-0.006376369

The left part of this table displays the effect of body mass index on potential mediators (IVW method) and the results of horizontal pleiotropy (egger_intercept). The right part of this table shows the effect of potential mediators on breast cancer(IVW method) and the results of horizontal pleiotropy (egger_intercept). The left part shows the effect of body mass index on a certain metabolic trait, and the right part shows the effect of this metabolic trait on breast cancer. The MR-Egger method was employed for detecting horizontal pleiotropy due to its allowance for the presence of non-zero intercepts (The columns in the table corresponding to “egger_intercept”, “se”, and “pval”), a pval below 0.05 suggests the existence of horizontal pleiotropy. BMI_IVW_Beta * BC_IVW_Beta represents the product of the Beta value of BMI on a potential mediator obtained from the IVW method and the Beta value of a potential mediator on breast cancer obtained from the IVW method. A negative value indicates that BMI reduces the risk of breast cancer through this mediator. Delta method: estimate the significance of the mediator effect (IVW_Beta_BMI×IVW_Beta_Breast). according to the z-table, Z-values falling between -1.96 and 1.96 are considered statistically insignificant.

#### 3.3.2 Analysis of metabolic characteristics mediating the association between BMI and ER+BC

**Figure 3:**
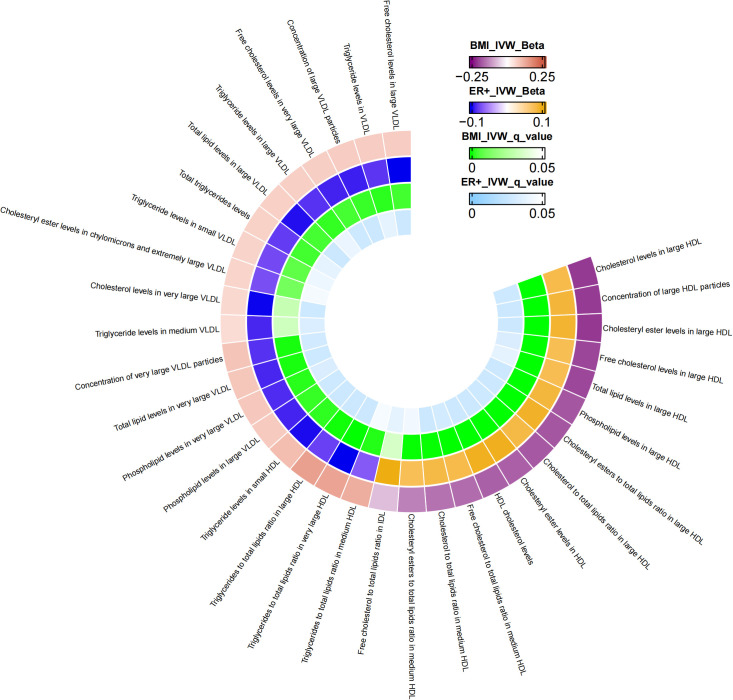
The mediator of 33 metabolic traits between BMI and ER+BC causality. We visualized that 33 metabolic traits mediate the effect of BMI on ER+breast cancer. The outermost boundary of the circular heat map consists of 33 metabolic traits and their corresponding identifiers. From the outermost to the innermost rings, the first and second rings of the circular heat map respectively depict the beta values of BMI on a particular metabolic trait and the beta values of the same trait on ER+ breast cancer (IVW method), while the third and fourth rings represent the q-values of these two aforementioned effects (IVW method).

Additional MR analysis was performed to investigate metabolic characteristics that mediate the association between BMI and ER+BC. **Supplementary Table S4** presents the MR analysis results of the impact of 249 metabolic traits on ER^+^BC. Preliminary analysis identified 75 metabolic traits exhibiting a causal effect on ER^+^ BC. Metabolic traits influencing ER^+^BC were selected based on FDR-adjusted p-values, resulting in the identification of 37 metabolic traits (**Supplementary Table S5**). Following FDR correction, mediator factors between BMI and ER^+^BC were identified, leading to the discovery of 33 metabolic traits with q-values below 0.05 (**Supplementary Table S8**). After excluding mediators with horizontal pleiotropy and those with q-values below 0.05 between BMI and ER^+^BC, the final results are shown in **Table 4**. A total of 33 metabolic traits mediate the causal relationship between BMI and ER^+^BC, predominantly involving various forms of cholesterol (particularly significant in HDL), triglyceride levels, concentrations of four lipoproteins/lipoproteins, and three phospholipids. **Figure 3** illustrates the mediation of 33 metabolic traits between BMI and ER^+^BC causality.

**Table 4:** The mediators between body mass index and ER+ breast cancer were screened according to their P-value (p<0.05) after the FDR corrected (mediators with horizontal pleiotropy were excluded).

	The impact of body mass index (id:ieu-a-974) on potential mediators										The impact of potential mediators on ER+ breast cancer (id:ieu-a-1127)											
	outcome	exposure	Number of SNPs	IVW_Beta	IVW_se	IVW_pval	IVW_q_value	egger_intercept	se	pval	outcome	exposure	Number of SNPs	IVW_Beta	IVW_se	IVW_pval	IVW_q_value	egger_intercept	se	pval	Z	BMI_IVW_Beta * ER+_IVW_Beta
1	HDL cholesterol levels || id:ebi-a-GCST90092822	Body mass index || id:ieu-a-974	34	-0.173062555	0.034780511	6.50E-07	3.30E-06	-0.003464685	0.003458168	0.32391551	ER+ Breast cancer (Combined Oncoarray; iCOGS; GWAS meta-analysis) || id:ieu-a-1127	HDL cholesterol levels || id:ebi-a-GCST90092822	72	0.096513735	0.028541853	0.000720962	0.026962563	0.003229478	0.00274147	0.242782142	-2.523251733	-0.016702914
2	Cholesteryl ester levels in HDL || id:ebi-a-GCST90092823	Body mass index || id:ieu-a-974	34	-0.173433271	0.03502089	7.33E-07	3.65E-06	-0.003309471	0.003487492	0.349755825	ER+ Breast cancer (Combined Oncoarray; iCOGS; GWAS meta-analysis) || id:ieu-a-1127	Cholesteryl ester levels in HDL || id:ebi-a-GCST90092823	76	0.0968154	0.028447005	0.000665625	0.026962563	0.002675736	0.002614448	0.309432947	-2.517757885	-0.016791012
3	Free cholesterol to total lipids ratio in IDL || id:ebi-a-GCST90092836	Body mass index || id:ieu-a-974	37	-0.069114589	0.030894472	0.025278611	0.035967853	-0.000135205	0.00310351	0.965498605	ER+ Breast cancer (Combined Oncoarray; iCOGS; GWAS meta-analysis) || id:ieu-a-1127	Free cholesterol to total lipids ratio in IDL || id:ebi-a-GCST90092836	49	0.099659457	0.035007099	0.004415616	0.03791339	-0.000275912	0.0031615	0.930825707	-1.683943888	-0.006887922
4	Cholesterol levels in large HDL || id:ebi-a-GCST90092844	Body mass index || id:ieu-a-974	33	-0.211846231	0.024722028	1.04E-17	8.65E-16	-0.003711157	0.002374461	0.128217192	ER+ Breast cancer (Combined Oncoarray; iCOGS; GWAS meta-analysis) || id:ieu-a-1127	Cholesterol levels in large HDL || id:ebi-a-GCST90092844	94	0.082057773	0.026626452	0.002057433	0.02747003	-9.22E-05	0.002323335	0.968442853	-3.063329056	-0.01738363
5	Cholesterol to total lipids ratio in large HDL || id:ebi-a-GCST90092845	Body mass index || id:ieu-a-974	35	-0.181553407	0.034643843	1.60E-07	1.29E-06	-0.002414184	0.00346177	0.490446756	ER+ Breast cancer (Combined Oncoarray; iCOGS; GWAS meta-analysis) || id:ieu-a-1127	Cholesterol to total lipids ratio in large HDL || id:ebi-a-GCST90092845	76	0.08400461	0.026992595	0.001857393	0.02747003	-0.001684577	0.002407131	0.486229095	-2.281098775	-0.015251323
6	Cholesteryl ester levels in large HDL || id:ebi-a-GCST90092846	Body mass index || id:ieu-a-974	33	-0.214121746	0.02510269	1.47E-17	9.12E-16	-0.003614961	0.002418553	0.145108841	ER+ Breast cancer (Combined Oncoarray; iCOGS; GWAS meta-analysis) || id:ieu-a-1127	Cholesteryl ester levels in large HDL || id:ebi-a-GCST90092846	95	0.091554879	0.026295345	0.000498074	0.026962563	0.000600898	0.002266276	0.791482479	-3.328585691	-0.019603891
7	Cholesteryl esters to total lipids ratio in large HDL || id:ebi-a-GCST90092847	Body mass index || id:ieu-a-974	35	-0.183038567	0.033628091	5.24E-08	5.43E-07	-0.001958767	0.003367782	0.564775409	ER+ Breast cancer (Combined Oncoarray; iCOGS; GWAS meta-analysis) || id:ieu-a-1127	Cholesteryl esters to total lipids ratio in large HDL || id:ebi-a-GCST90092847	69	0.09392625	0.028920625	0.001163314	0.026962563	-0.000821445	0.002564134	0.749692608	-2.555314973	-0.017192126
8	Free cholesterol levels in large HDL || id:ebi-a-GCST90092848	Body mass index || id:ieu-a-974	33	-0.200195714	0.023362377	1.04E-17	8.65E-16	-0.004020638	0.002216042	0.079315506	ER+ Breast cancer (Combined Oncoarray; iCOGS; GWAS meta-analysis) || id:ieu-a-1127	Free cholesterol levels in large HDL || id:ebi-a-GCST90092848	81	0.078386338	0.026442594	0.003032771	0.032371928	0.000837454	0.002437892	0.732123054	-3.067501867	-0.015692609
9	Total lipid levels in large HDL || id:ebi-a-GCST90092850	Body mass index || id:ieu-a-974	33	-0.199036508	0.024246616	2.23E-16	1.11E-14	-0.004149204	0.002301235	0.081111832	ER+ Breast cancer (Combined Oncoarray; iCOGS; GWAS meta-analysis) || id:ieu-a-1127	Total lipid levels in large HDL || id:ebi-a-GCST90092850	85	0.078968622	0.027372909	0.003915145	0.037495044	0.000472414	0.002456807	0.847986631	-2.972349527	-0.015717639
10	Concentration of large HDL particles || id:ebi-a-GCST90092851	Body mass index || id:ieu-a-974	33	-0.210683268	0.023819901	9.17E-19	2.28E-16	-0.0039512	0.002267858	0.091377589	ER+ Breast cancer (Combined Oncoarray; iCOGS; GWAS meta-analysis) || id:ieu-a-1127	Concentration of large HDL particles || id:ebi-a-GCST90092851	87	0.088035762	0.026154698	0.000762768	0.026962563	0.002235586	0.002327653	0.339554831	-3.359189891	-0.018547662
11	Phospholipid levels in large HDL || id:ebi-a-GCST90092852	Body mass index || id:ieu-a-974	33	-0.183567261	0.024583683	8.20E-14	2.04E-12	-0.004357706	0.002324307	0.070263509	ER+ Breast cancer (Combined Oncoarray; iCOGS; GWAS meta-analysis) || id:ieu-a-1127	Phospholipid levels in large HDL || id:ebi-a-GCST90092852	75	0.086891734	0.027212592	0.001407684	0.026962563	0.001743091	0.002578324	0.501141599	-3.130790818	-0.015950478
12	Triglycerides to total lipids ratio in large HDL || id:ebi-a-GCST90092855	Body mass index || id:ieu-a-974	37	0.145148989	0.03851432	0.000164099	0.00037146	0.004688692	0.003786039	0.223804034	ER+ Breast cancer (Combined Oncoarray; iCOGS; GWAS meta-analysis) || id:ieu-a-1127	Triglycerides to total lipids ratio in large HDL || id:ebi-a-GCST90092855	66	-0.081442966	0.025466353	0.001383547	0.026962563	-0.00233029	0.002618358	0.376809039	-1.982565261	-0.011821364
13	Free cholesterol levels in large VLDL || id:ebi-a-GCST90092872	Body mass index || id:ieu-a-974	37	0.076359311	0.02474134	0.002026611	0.003528855	0.003628415	0.002408516	0.140913601	ER+ Breast cancer (Combined Oncoarray; iCOGS; GWAS meta-analysis) || id:ieu-a-1127	Free cholesterol levels in large VLDL || id:ebi-a-GCST90092872	54	-0.100730199	0.030145328	0.000833311	0.026962563	-0.000791037	0.003169909	0.803921785	-2.150749344	-0.007691689
14	Total lipid levels in large VLDL || id:ebi-a-GCST90092874	Body mass index || id:ieu-a-974	37	0.071556431	0.023482238	0.002309396	0.003938627	0.002834272	0.002309797	0.227988122	ER+ Breast cancer (Combined Oncoarray; iCOGS; GWAS meta-analysis) || id:ieu-a-1127	Total lipid levels in large VLDL || id:ebi-a-GCST90092874	59	-0.097545662	0.029820453	0.001071303	0.026962563	-0.002098623	0.002892756	0.471128721	-2.077822292	-0.006980019
15	Concentration of large VLDL particles || id:ebi-a-GCST90092875	Body mass index || id:ieu-a-974	37	0.075302748	0.023574035	0.001401661	0.002566276	0.002964418	0.002314538	0.208692614	ER+ Breast cancer (Combined Oncoarray; iCOGS; GWAS meta-analysis) || id:ieu-a-1127	Concentration of large VLDL particles || id:ebi-a-GCST90092875	59	-0.093639061	0.029167456	0.001325526	0.026962563	-0.001621148	0.002934357	0.582785422	-2.164678093	-0.007051279
16	Phospholipid levels in large VLDL || id:ebi-a-GCST90092876	Body mass index || id:ieu-a-974	37	0.081157023	0.025262376	0.001315554	0.002426466	0.003620578	0.002462826	0.150470237	ER+ Breast cancer (Combined Oncoarray; iCOGS; GWAS meta-analysis) || id:ieu-a-1127	Phospholipid levels in large VLDL || id:ebi-a-GCST90092876	59	-0.091977245	0.0297885	0.002017281	0.02747003	-0.000670971	0.003021285	0.825044303	-2.181339892	-0.007464599
17	Triglyceride levels in large VLDL || id:ebi-a-GCST90092878	Body mass index || id:ieu-a-974	37	0.072680165	0.022316042	0.001126531	0.002125047	0.002089648	0.002213864	0.351695451	ER+ Breast cancer (Combined Oncoarray; iCOGS; GWAS meta-analysis) || id:ieu-a-1127	Triglyceride levels in large VLDL || id:ebi-a-GCST90092878	59	-0.086869392	0.031033575	0.005122832	0.040446079	-0.0016452	0.002991413	0.584485808	-2.00678481	-0.006313682
18	Cholesterol to total lipids ratio in medium HDL || id:ebi-a-GCST90092893	Body mass index || id:ieu-a-974	36	-0.152662441	0.03735168	4.37E-05	0.000112333	-0.004337578	0.00366462	0.244766747	ER+ Breast cancer (Combined Oncoarray; iCOGS; GWAS meta-analysis) || id:ieu-a-1127	Cholesterol to total lipids ratio in medium HDL || id:ebi-a-GCST90092893	78	0.084449924	0.026438971	0.001402453	0.026962563	0.000410849	0.002580085	0.873903275	-2.105299738	-0.012892332
19	Cholesteryl esters to total lipids ratio in medium HDL || id:ebi-a-GCST90092895	Body mass index || id:ieu-a-974	37	-0.136397048	0.033629532	4.99E-05	0.00012437	-0.003482782	0.003326241	0.30224812	ER+ Breast cancer (Combined Oncoarray; iCOGS; GWAS meta-analysis) || id:ieu-a-1127	Cholesteryl esters to total lipids ratio in medium HDL || id:ebi-a-GCST90092895	71	0.07719343	0.028056986	0.005935767	0.043470768	0.000586845	0.002832343	0.83646845	-2.075662089	-0.010528956
20	Free cholesterol to total lipids ratio in medium HDL || id:ebi-a-GCST90092897	Body mass index || id:ieu-a-974	34	-0.159565519	0.033954669	2.61E-06	1.02E-05	-0.003983125	0.003355604	0.243962328	ER+ Breast cancer (Combined Oncoarray; iCOGS; GWAS meta-analysis) || id:ieu-a-1127	Free cholesterol to total lipids ratio in medium HDL || id:ebi-a-GCST90092897	75	0.081972938	0.027225211	0.002604572	0.029479022	0.001533381	0.002702374	0.572169691	-2.2322315	-0.013080054
21	Triglycerides to total lipids ratio in medium HDL || id:ebi-a-GCST90092903	Body mass index || id:ieu-a-974	37	0.123780062	0.035964667	0.000578043	0.001170185	0.004437306	0.003533479	0.217511109	ER+ Breast cancer (Combined Oncoarray; iCOGS; GWAS meta-analysis) || id:ieu-a-1127	Triglycerides to total lipids ratio in medium HDL || id:ebi-a-GCST90092903	67	-0.07076483	0.026110415	0.006723966	0.046001647	-0.000722848	0.00287014	0.801950824	-1.81730427	-0.008759275
22	Triglyceride levels in medium VLDL || id:ebi-a-GCST90092926	Body mass index || id:ieu-a-974	37	0.052254431	0.023077134	0.023553336	0.033705636	0.001803148	0.002298134	0.437958634	ER+ Breast cancer (Combined Oncoarray; iCOGS; GWAS meta-analysis) || id:ieu-a-1127	Triglyceride levels in medium VLDL || id:ebi-a-GCST90092926	57	-0.090952132	0.030945778	0.003291876	0.032787085	-0.001532475	0.00306127	0.618649296	-1.552123646	-0.004752652
23	Triglyceride levels in small HDL || id:ebi-a-GCST90092954	Body mass index || id:ieu-a-974	37	0.100675652	0.030480432	0.0009567	0.001832449	0.004031473	0.002984989	0.185497634	ER+ Breast cancer (Combined Oncoarray; iCOGS; GWAS meta-analysis) || id:ieu-a-1127	Triglyceride levels in small HDL || id:ebi-a-GCST90092954	63	-0.098305615	0.026803614	0.000244814	0.026962563	-0.004096132	0.002962714	0.171840525	-2.446910577	-0.009896982
24	Triglyceride levels in small VLDL || id:ebi-a-GCST90092978	Body mass index || id:ieu-a-974	37	0.067175142	0.023603855	0.004428061	0.007206452	0.001549378	0.002356623	0.515187861	ER+ Breast cancer (Combined Oncoarray; iCOGS; GWAS meta-analysis) || id:ieu-a-1127	Triglyceride levels in small VLDL || id:ebi-a-GCST90092978	72	-0.07745514	0.027899503	0.005499509	0.041496297	-0.000626056	0.002629733	0.8125237	-1.941244283	-0.00520306
25	Total triglycerides levels || id:ebi-a-GCST90092992	Body mass index || id:ieu-a-974	37	0.070282804	0.022789316	0.002042209	0.00353132	0.001705823	0.002271135	0.457620132	ER+ Breast cancer (Combined Oncoarray; iCOGS; GWAS meta-analysis) || id:ieu-a-1127	Total triglycerides levels || id:ebi-a-GCST90092992	63	-0.08336518	0.029016675	0.00406582	0.0374959	-0.000195239	0.002891196	0.946381491	-2.0195644	-0.005859139
26	Triglyceride levels in VLDL || id:ebi-a-GCST90093003	Body mass index || id:ieu-a-974	37	0.075888196	0.022581284	0.000777543	0.001548865	0.002050699	0.002241845	0.366588931	ER+ Breast cancer (Combined Oncoarray; iCOGS; GWAS meta-analysis) || id:ieu-a-1127	Triglyceride levels in VLDL || id:ebi-a-GCST90093003	61	-0.085452321	0.02997724	0.004364049	0.03791339	-0.001251437	0.002899902	0.667645305	-2.104117313	-0.006484822
27	Triglycerides to total lipids ratio in very large HDL || id:ebi-a-GCST90093015	Body mass index || id:ieu-a-974	37	0.139560841	0.032175611	1.44E-05	4.49E-05	0.00441667	0.003144588	0.168973719	ER+ Breast cancer (Combined Oncoarray; iCOGS; GWAS meta-analysis) || id:ieu-a-1127	Triglycerides to total lipids ratio in very large HDL || id:ebi-a-GCST90093015	62	-0.101028125	0.02718319	0.000201949	0.026962563	-0.00238563	0.003040304	0.435735419	-2.678660213	-0.01409957
28	Cholesterol levels in very large VLDL || id:ebi-a-GCST90093016	Body mass index || id:ieu-a-974	37	0.058601199	0.025474268	0.02142514	0.031016627	0.004016278	0.002467298	0.112536591	ER+ Breast cancer (Combined Oncoarray; iCOGS; GWAS meta-analysis) || id:ieu-a-1127	Cholesterol levels in very large VLDL || id:ebi-a-GCST90093016	53	-0.099461226	0.032331802	0.002096107	0.02747003	-0.000404156	0.003442249	0.906996001	-1.643989207	-0.005828547
29	Free cholesterol levels in very large VLDL || id:ebi-a-GCST90093020	Body mass index || id:ieu-a-974	37	0.074265467	0.024352072	0.002291029	0.003934249	0.003398832	0.002377899	0.161771377	ER+ Breast cancer (Combined Oncoarray; iCOGS; GWAS meta-analysis) || id:ieu-a-1127	Free cholesterol levels in very large VLDL || id:ebi-a-GCST90093020	56	-0.092176702	0.02935665	0.001690103	0.02747003	-0.001422536	0.002913672	0.627365087	-2.103270489	-0.006845546
30	Total lipid levels in very large VLDL || id:ebi-a-GCST90093022	Body mass index || id:ieu-a-974	37	0.087178533	0.023983503	0.000278048	0.000602034	0.003320101	0.002343057	0.165324673	ER+ Breast cancer (Combined Oncoarray; iCOGS; GWAS meta-analysis) || id:ieu-a-1127	Total lipid levels in very large VLDL || id:ebi-a-GCST90093022	58	-0.090960822	0.029376044	0.001958671	0.02747003	-0.001802524	0.002999425	0.55029366	-2.337201842	-0.007929831
31	Concentration of very large VLDL particles || id:ebi-a-GCST90093023	Body mass index || id:ieu-a-974	37	0.089416778	0.024488553	0.000260845	0.00056974	0.003495901	0.002388017	0.152136127	ER+ Breast cancer (Combined Oncoarray; iCOGS; GWAS meta-analysis) || id:ieu-a-1127	Concentration of very large VLDL particles || id:ebi-a-GCST90093023	61	-0.0872507	0.02878753	0.002438659	0.028915531	-0.000412209	0.002894565	0.88724297	-2.341301852	-0.007801676
32	Phospholipid levels in very large VLDL || id:ebi-a-GCST90093024	Body mass index || id:ieu-a-974	37	0.083345498	0.024602211	0.000704752	0.001415187	0.003432086	0.002402401	0.161981484	ER+ Breast cancer (Combined Oncoarray; iCOGS; GWAS meta-analysis) || id:ieu-a-1127	Phospholipid levels in very large VLDL || id:ebi-a-GCST90093024	58	-0.089220199	0.030186398	0.003120186	0.032371928	-0.001035811	0.003046385	0.735118737	-2.196801542	-0.007436102
33	Cholesteryl ester levels in chylomicrons and extremely large VLDL || id:ebi-a-GCST90093042	Body mass index || id:ieu-a-974	37	0.065180053	0.024075115	0.006782025	0.010894994	0.004213153	0.002311299	0.076877747	ER+ Breast cancer (Combined Oncoarray; iCOGS; GWAS meta-analysis) || id:ieu-a-1127	Cholesteryl ester levels in chylomicrons and extremely large VLDL || id:ebi-a-GCST90093042	62	-0.075179335	0.027530152	0.006318082	0.04494864	-0.001301842	0.002771892	0.640301686	-1.886631038	-0.004900193

The left part of the table displays the effect of body mass index on potential mediators (IVW method) and the results of horizontal pleiotropy (egger_intercept). The right part of the table shows the effect of potential mediators on ER+ breast cancer(IVW method) and the results of horizontal pleiotropy (egger_intercept). The left part shows the effect of body mass index on a certain metabolic trait, and the right part shows the effect of this metabolic trait on ER+ breast cancer. The MR-Egger method is employed for detecting horizontal pleiotropy due to its allowance for the presence of non-zero intercepts (The columns in the table corresponding to “egger_intercept”, “se”, and “pval”), a pval below 0.05 suggests the existence of horizontal pleiotropy. BMI_IVW_Beta * ER+_IVW_Beta represents the product of the Beta value of BMI on a potential mediator obtained from the IVW method and the Beta value of a potential mediator on ER+ breast cancer obtained from the IVW method. A negative value indicates that BMI reduces the risk of breast cancer through this mediator. Delta method: estimate the significance of the mediator effect (BMI_IVW_Beta * ER+_IVW_Beta). according to the z-table, Z-values falling between -1.96 and 1.96 are considered statistically insignificant.

#### 3.3.3 Analysis of metabolic traits mediating the association between BMI and ER^-^BC

**Figure 4:**
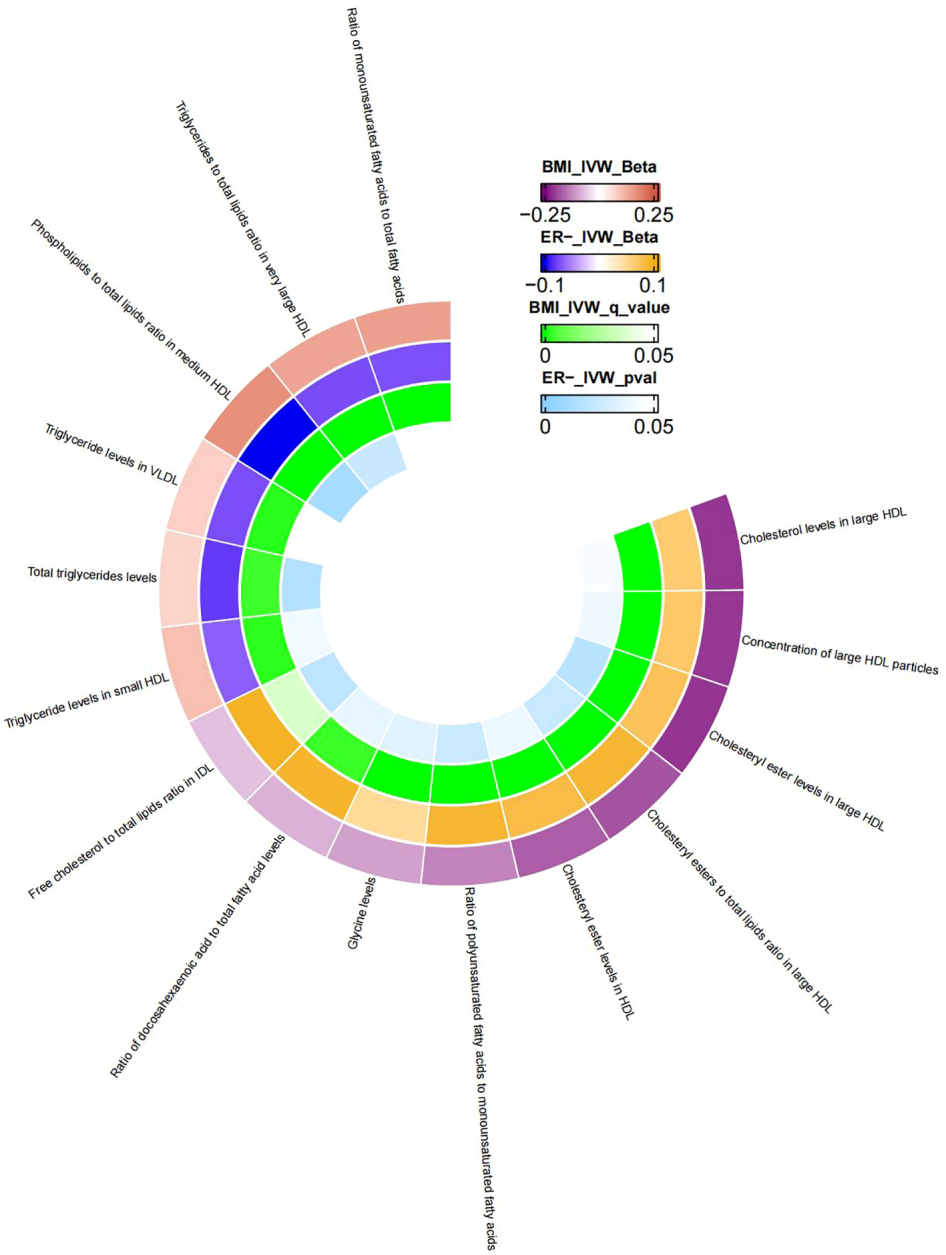
The mediator of 15 metabolic traits between BMI and ER-BC causality. We visualized that 15 metabolic traits mediate the effect of BMI on ER-breast cancer. The outermost boundary of the circular heat map consists of 15 metabolic traits and their corresponding identifiers. From the outermost to the innermost rings, the first and second rings of the circular heat map respectively depict the beta values of BMI on a particular metabolic trait and the beta values of the same trait on ER- breast cancer (IVW method), while the third and fourth rings represent the q-values of these two aforementioned effects (IVW method).

Similarly, MR analysis was performed to explore metabolic traits mediating the relationship between BMI and ER^-^BC. The **Supplementary Table S6** presents the MR analysis results of the impact of 249 metabolic traits on ER^-^BC. Mediators between BMI and ER^-^BC were screened based on their P-values before FDR correction (**Supplementary Table S9**). Following FDR correction, no statistically significant results were observed. Therefore, potential mediators were screened using uncorrected p-values. The final results, presented in **Table 5**, reveal 15 metabolic traits mediating the causal relationship between BMI and ER^-^ BC, mainly involving various forms of cholesterol (especially in HDL), triglyceride levels, concentrations of large HDL particles, and large HDL particle concentrations. Additionally, glycine, two unsaturated fatty acids (docosahexaenoic acid and monounsaturated fatty acid), and the r relative proportion of polyunsaturated fatty acids to monounsaturated fatty acids also mediate the causal association between BMI and ER^-^BC. **Figure 4** illustrates the mediation of 15 metabolic traits between BMI and ER^-^BC causality.

**Table 5:** The mediators between body mass index and ER- breast cancer were screened according to their P-value (p<0.05) before FDR corrected.

	The impact of body mass index (id:ieu-a-974) on potential mediators										The impact of potential mediators on ER- breast cancer (id:ieu-a-1128)											
	outcome	exposure	Number of SNPs	IVW_Beta	IVW_se	IVW_pval	IVW_q_value	egger_intercept	se	pval	outcome	exposure	Number of SNPs	IVW_Beta	IVW_se	IVW_pval	IVW_q_value	egger_intercept	se	pval	Z	BMI_IVW_Beta * ER-_IVW_Beta
1	Ratio of docosahexaenoic acid to total fatty acid levels || id:ebi-a-GCST90092817	Body mass index || id:ieu-a-974	37	-0.085086449	0.027155212	0.001728305	0.003073914	-0.003304708	0.002670168	0.224087977	ER- Breast cancer (Combined Oncoarray; iCOGS; GWAS meta-analysis) || id:ieu-a-1128	Ratio of docosahexaenoic acid to total fatty acid levels || id:ebi-a-GCST90092817	28	0.091509172	0.043974997	0.037439727	0.561723713	-0.000929276	0.004886817	0.850660886	-1.677960732	-0.00778619
2	Glycine levels || id:ebi-a-GCST90092820	Body mass index || id:ieu-a-974	37	-0.10282217	0.021862485	2.56E-06	1.01E-05	0.001608154	0.002179492	0.465519909	ER- Breast cancer (Combined Oncoarray; iCOGS; GWAS meta-analysis) || id:ieu-a-1128	Glycine levels || id:ebi-a-GCST90092820	40	0.047285359	0.022330825	0.034218052	0.561723713	-0.001322072	0.002817981	0.641639042	-1.957639942	-0.004861983
3	Cholesteryl ester levels in HDL || id:ebi-a-GCST90092823	Body mass index || id:ieu-a-974	34	-0.173433271	0.03502089	7.33E-07	3.65E-06	-0.003309471	0.003487492	0.349755825	ER- Breast cancer (Combined Oncoarray; iCOGS; GWAS meta-analysis) || id:ieu-a-1128	Cholesteryl ester levels in HDL || id:ebi-a-GCST90092823	76	0.082791041	0.040150917	0.039208093	0.561723713	0.003861805	0.003687104	0.298332557	-2.073782585	-0.014358721
4	Free cholesterol to total lipids ratio in IDL || id:ebi-a-GCST90092836	Body mass index || id:ieu-a-974	37	-0.069114589	0.030894472	0.025278611	0.035967853	-0.000135205	0.00310351	0.965498605	ER- Breast cancer (Combined Oncoarray; iCOGS; GWAS meta-analysis) || id:ieu-a-1128	Free cholesterol to total lipids ratio in IDL || id:ebi-a-GCST90092836	49	0.09402693	0.040339359	0.019758741	0.561723713	-0.003253847	0.003615251	0.372691076	-1.493008824	-0.006498633
5	Cholesterol levels in large HDL || id:ebi-a-GCST90092844	Body mass index || id:ieu-a-974	33	-0.211846231	0.024722028	1.04E-17	8.65E-16	-0.003711157	0.002374461	0.128217192	ER- Breast cancer (Combined Oncoarray; iCOGS; GWAS meta-analysis) || id:ieu-a-1128	Cholesterol levels in large HDL || id:ebi-a-GCST90092844	94	0.065129434	0.032386192	0.044322844	0.561723713	-0.000840383	0.002822204	0.766545961	-2.443722075	-0.013797425
6	Cholesteryl ester levels in large HDL || id:ebi-a-GCST90092846	Body mass index || id:ieu-a-974	33	-0.214121746	0.02510269	1.47E-17	9.12E-16	-0.003614961	0.002418553	0.145108841	ER- Breast cancer (Combined Oncoarray; iCOGS; GWAS meta-analysis) || id:ieu-a-1128	Cholesteryl ester levels in large HDL || id:ebi-a-GCST90092846	95	0.075590332	0.032005456	0.018186688	0.561723713	0.000169479	0.002758236	0.951136992	-2.745915426	-0.016185534
7	Cholesteryl esters to total lipids ratio in large HDL || id:ebi-a-GCST90092847	Body mass index || id:ieu-a-974	35	-0.183038567	0.033628091	5.24E-08	5.43E-07	-0.001958767	0.003367782	0.564775409	ER- Breast cancer (Combined Oncoarray; iCOGS; GWAS meta-analysis) || id:ieu-a-1128	Cholesteryl esters to total lipids ratio in large HDL || id:ebi-a-GCST90092847	69	0.08922574	0.039608775	0.024279581	0.561723713	0.002868	0.003491915	0.414373817	-2.301000653	-0.016331752
8	Concentration of large HDL particles || id:ebi-a-GCST90092851	Body mass index || id:ieu-a-974	33	-0.210683268	0.023819901	9.17E-19	2.28E-16	-0.0039512	0.002267858	0.091377589	ER- Breast cancer (Combined Oncoarray; iCOGS; GWAS meta-analysis) || id:ieu-a-1128	Concentration of large HDL particles || id:ebi-a-GCST90092851	87	0.069087353	0.033676737	0.040219647	0.561723713	0.000560428	0.003008939	0.852688989	-2.631365618	-0.014555549
9	Phospholipids to total lipids ratio in medium HDL || id:ebi-a-GCST90092901	Body mass index || id:ieu-a-974	34	0.170200915	0.033523685	3.83E-07	2.39E-06	0.002192949	0.003363125	0.519020883	ER- Breast cancer (Combined Oncoarray; iCOGS; GWAS meta-analysis) || id:ieu-a-1128	Phospholipids to total lipids ratio in medium HDL || id:ebi-a-GCST90092901	74	-0.105247809	0.041571684	0.011350501	0.561723713	0.000643638	0.003883769	0.868837702	-2.491339754	-0.017913273
10	Ratio of monounsaturated fatty acids to total fatty acids || id:ebi-a-GCST90092929	Body mass index || id:ieu-a-974	37	0.147290827	0.029238512	4.71E-07	2.67E-06	0.004931765	0.002816395	0.088691087	ER- Breast cancer (Combined Oncoarray; iCOGS; GWAS meta-analysis) || id:ieu-a-1128	Ratio of monounsaturated fatty acids to total fatty acids || id:ebi-a-GCST90092929	61	-0.073292722	0.037001943	0.04761591	0.561723713	0.00343941	0.003609895	0.344592297	-2.121171564	-0.010795346
11	Ratio of polyunsaturated fatty acids to monounsaturated fatty acids || id:ebi-a-GCST90092940	Body mass index || id:ieu-a-974	37	-0.132481572	0.028274045	2.79E-06	1.07E-05	-0.004627115	0.002730537	0.099036462	ER- Breast cancer (Combined Oncoarray; iCOGS; GWAS meta-analysis) || id:ieu-a-1128	Ratio of polyunsaturated fatty acids to monounsaturated fatty acids || id:ebi-a-GCST90092940	53	0.089878071	0.040032576	0.024760214	0.561723713	0.000558915	0.003987194	0.889071855	-2.292519887	-0.011907188
12	Triglyceride levels in small HDL || id:ebi-a-GCST90092954	Body mass index || id:ieu-a-974	37	0.100675652	0.030480432	0.0009567	0.001832449	0.004031473	0.002984989	0.185497634	ER- Breast cancer (Combined Oncoarray; iCOGS; GWAS meta-analysis) || id:ieu-a-1128	Triglyceride levels in small HDL || id:ebi-a-GCST90092954	63	-0.067687141	0.03317738	0.041334459	0.561723713	-0.00268831	0.003696587	0.469860299	-1.792058299	-0.006814447
13	Total triglycerides levels || id:ebi-a-GCST90092992	Body mass index || id:ieu-a-974	37	0.070282804	0.022789316	0.002042209	0.00353132	0.001705823	0.002271135	0.457620132	ER- Breast cancer (Combined Oncoarray; iCOGS; GWAS meta-analysis) || id:ieu-a-1128	Total triglycerides levels || id:ebi-a-GCST90092992	63	-0.082380519	0.034045418	0.015532287	0.561723713	-0.003607102	0.003347716	0.285507887	-1.792655809	-0.005789934
14	Triglyceride levels in VLDL || id:ebi-a-GCST90093003	Body mass index || id:ieu-a-974	37	0.075888196	0.022581284	0.000777543	0.001548865	0.002050699	0.002241845	0.366588931	ER- Breast cancer (Combined Oncoarray; iCOGS; GWAS meta-analysis) || id:ieu-a-1128	Triglyceride levels in VLDL || id:ebi-a-GCST90093003	61	-0.074643114	0.03798932	0.049432216	0.561723713	-0.00219581	0.003649829	0.549731991	-1.709672427	-0.005664531
15	Triglycerides to total lipids ratio in very large HDL || id:ebi-a-GCST90093015	Body mass index || id:ieu-a-974	37	0.139560841	0.032175611	1.44E-05	4.49E-05	0.00441667	0.003144588	0.168973719	ER- Breast cancer (Combined Oncoarray; iCOGS; GWAS meta-analysis) || id:ieu-a-1128	Triglycerides to total lipids ratio in very large HDL || id:ebi-a-GCST90093015	62	-0.074768727	0.033066903	0.023750908	0.561723713	0.000290243	0.003709974	0.937903007	-2.035622767	-0.010434786

After applying False Discovery Rate (FDR) correction, no statistically significant results were observed. Therefore, we proceeded to screen for potential mediators using the uncorrected p-values. The left part of the table displays the effect of body mass index on potential mediators (IVW method) and the results of horizontal pleiotropy (egger_intercept). The right part of the table shows the effect of potential mediators on ER- breast cancer(IVW method) and the results of horizontal pleiotropy (egger_intercept). The left part shows the effect of body mass index on a certain metabolic trait, and the right part shows the effect of this metabolic trait on ER- breast cancer. The MR-Egger method is employed for detecting horizontal pleiotropy due to its allowance for the presence of non-zero intercepts (The columns in the table corresponding to “egger_intercept”, “se”, and “pval”), a pval below 0.05 suggests the existence of horizontal pleiotropy. BMI_IVW_Beta * ER-_IVW_Beta represents the product of the Beta value of BMI on a potential mediator obtained from the IVW method and the Beta value of a potential mediator on ER- breast cancer obtained from the IVW method. A negative value indicates that BMI reduces the risk of breast cancer through this mediator. Delta method: estimate the significance of the mediator effect (BMI_IVW_Beta * ER-_IVW_Beta). according to the z-table, Z-values falling between -1.96 and 1.96 are considered statistically insignificant.

It is noteworthy to mention that in the MR analysis of BC, ER^+^BC, and ER^-^BC, the causal relationship between BMI and metabolites is not consistent. For example, in the causal analysis and mediation analysis of BMI and BC, BMI has a negative causal relationship with most cholesterol in HDL but a positive causal relationship with cholesterol in VLDL and triglyceride levels; correspondingly, cholesterol in HDL is positively associated with BC risk, while cholesterol in VLDL and triglyceride levels are negatively associated with BC in MR analysis results. Therefore, BMI has an effect on metabolites, but it is not simply an increase or decrease; it has different effects on metabolites in different lipoproteins (HDL, IDL, VLDL, etc.). Similarly, the effects of metabolites in different lipoproteins (HDL, IDL, VLDL, etc.) on BC are also different.

## 4 Discussion

Previous studies exploring the correlation between BMI and the risk of developing BC using observational research methodologies may be prone to inherent limitations. Our Mendelian randomization study indicates that genetically predicted BMI is linked to a heightened/decreased risk of BC. Furthermore, sex hormones and various metabolites assume significant mediating roles in the causal pathway from BMI to B. We provide causal evidence demonstrating that BMI increases ER^+^BC risk through bioavailable testosterone levels, while it decreases BC risk through multiple metabolite pathways (mostly various forms of cholesterol, triglyceride levels, etc.). However, this study was conducted at the genetic level, so these results should be interpreted with caution.

In previous studies, significant associations between BMI and several cancers, including colorectal cancer, esophageal cancer, liver cancer, gallbladder cancer, pancreatic cancer, etc., have been observed, showing a positive dose-response relationship (24). However, the impact of BMI on BC is not uniformly established (24). For instance, high BMI during childhood, adolescence, and early adulthood is linked to a diminished risk of premenopausal BC (25–27). Conversely, several investigations have identified an association between adult BMI and postmenopausal BC, particularly in cases of estrogen receptor-positive tumors. Notably, a consistent negative correlation has been observed regarding premenopausal BC (24, 28). High BMI is a well-known risk factor for postmenopausal women with ER^+^BC. This aligns with our study findings (28–30). We found that BMI appears to affect BC risk through two different pathways. In the sex hormone pathway, we found that elevated BMI is positively associated with increased bioavailable testosterone levels, which in turn increases the risk of ER+ BC. In contrast, in the metabolite pathway study, multiple metabolites mediate the negative causal relationship between BMI and BC, regardless of ER status. Thus, we speculate that premenopausal BMI primarily reduces BC risk through the metabolite pathway, while postmenopausal BMI increases ER^+^BC risk through the sex hormone pathway. However, this requires further validation through more literature and research.

Our study found that increased BMI is closely associated with elevated levels of bioavailable testosterone and reduced levels of SHBG. Furthermore, higher levels of both estrogen and testosterone have been associated with an increased risk of ER+ BC. These findings are consistent with previous studies (31, 32). A prospective study in premenopausal women demonstrated that elevated total and free estradiol levels, along with higher plasma total and free testosterone levels, were linked to an increased risk of BC (33). These associations appeared to be stronger in women with invasive BC or ER+/PR+ tumors and were independent of other known breast cancer risk factors. ER+BC is one of the more common subtypes and is highly dependent on estrogen. The potential mechanisms connecting testosterone to ER+ breast cancer likely involve its conversion to estradiol (34). Testosterone can be aromatized into estrogen, and estradiol binds to ER, inducing the transcription of growth-promoting genes while downregulating negative growth regulators, ultimately enhancing breast cancer cell proliferation (35). The interaction between high BMI and ER^+^BC can be partially explained by increased estrogen biosynthesis in adipose tissue (36, 37)and the creation of a pro-tumor environment through the release of various cytokines (38), induction of hypoxia-inducible factor changes (39), and triggering inflammation (40). Additionally, high BMI may increase the sensitivity of breast cells to insulin, leading to excessive production of insulin antibodies, resulting in a high insulin state, ultimately leading to lipid metabolism disorders (41).

The increase in BMI correlates with alterations in the levels of certain metabolites, as high BMI can cause an increase in adipose tissue, leading to metabolic abnormalities. Our study results show that BMI is negatively associated with multiple forms of cholesterol, while in the causality analysis between cholesterol and BC, high cholesterol is linked to a heightened risk of BC. There is evidence to suggest that hypercholesterolemia is considered an autonomous risk factor for postmenopausal women with BC (42). In a recent study, Wen Liu, Binita Chakraborty, et al. (43), found that prolonged exposure to 27-hydroxycholesterol (a major metabolite of cholesterol) selects cells that survive with increased cell uptake and/or lipid biosynthesis. These cells overexpress the iron death negative regulatory factor GPX4, demonstrating stronger tumorigenic and metastatic capabilities. Moreover, several studies (44, 45) have shown that 27-hydroxycholesterol can act as a true endogenous selective estrogen receptor modulator (SERM), thereby promoting the growth of ER-positive BC in luminal BC models. Therefore, high BMI reduces the generation of various forms of cholesterol, and the reduction in cholesterol generation weakens its relationship with the risk of BC. Among the various triglyceride-related metabolites, BMI shows a positive correlation with them, and correspondingly, these metabolites are linked to a diminished risk of BC, although the mechanism behind this is currently unclear. Overall, the relationship between BMI and metabolites is complex and diverse, and different metabolites may have different effects on the risk of BC (46).

Our study results provide a new perspective on early screening and prevention of BC, for populations with high BMI, different prevention strategies can be formulated based on ER status and menopausal status. Postmenopausal women with severe obesity may need to consider the potential risk of BC and take timely intervention measures. For individuals with a high BMI, the decision to pursue weight reduction should be based on the levels of monitored metabolites, such as triglycerides and cholesterol, in order to reduce the risk of breast cancer. Regular lipid monitoring is recommended for individuals with metabolic abnormalities, and appropriate pharmacological treatment may be considered for those with hypercholesterolemia. Multiple large studies (47, 48) have shown that the use of statins (a class of drugs that lower cholesterol) before or after BC diagnosis has significant benefits for overall survival and disease-free survival.

Our study has several strengths. Firstly, the structure of the MR model safeguards against the impact of confounding variables, thereby securing dependable estimations of causal effects derived from observational investigations (49). Additionally, the MR model utilizes extensive-sample Genome-Wide Association Studies (GWAS) datasets, substantially enhancing the analytical efficacy in comparison to small-sample models relying on individual-level data (50). To guarantee the accuracy of the MR analysis, we executed a thorough investigation into pleiotropy. Additionally, we utilized exposure and outcome data sourced from diverse European populations across multiple countries to mitigate potential biases.

Undoubtedly, this study is subject to certain limitations. Firstly, due to the lack of aggregated GWAS data for pre-and postmenopausal women, we were unable to stratify the BC population based on menopausal status. As a result, we analyzed pre-and postmenopausal women as a single group. Given the differences in sex hormone levels between pre- and postmenopausal women, further subgroup analyses are needed to clarify the specific populations in which BMI influences BC risk through bioavailable testosterone. Secondly, given that the data exclusively originate from individuals of European descent, the applicability of the results to other racial or ethnic groups may be limited. Finally, owing to incomplete GWAS data regarding specific exposures, we refrained from conducting reverse MR analysis. Thus, completing bidirectional MR analysis becomes imperative upon meeting future data prerequisites.

## 5 Conclusion

In this study, MR analysis revealed that higher BMI decreases the risk of BC, regardless of ER^+^ or ER^-^ status. Additionally, BMI may increase the likelihood of ER^+^BC through the pathway of sex hormones (bioavailable testosterone) and decrease the risk of BC through various metabolic pathways. These findings are of significant importance for developing preventive strategies and interventions targeting BC.

## Data Availability

Publicly available datasets were analyzed in this study. All GWAS data used in this study are available in the IEU open GWAS project (https://gwas.mrcieu.ac.uk/).
