# Supplementary figures and images for: Mediation effects of metabolites and sex hormones on the relationship between body mass index and breast cancer: Mendelian randomization analysis and mediation analysis
Source: Front Oncol. 2024 Nov 21;14:1449956. doi: 10.3389/fonc.2024.1449956 (PMC11617525; doi:10.3389/fonc.2024.1449956)

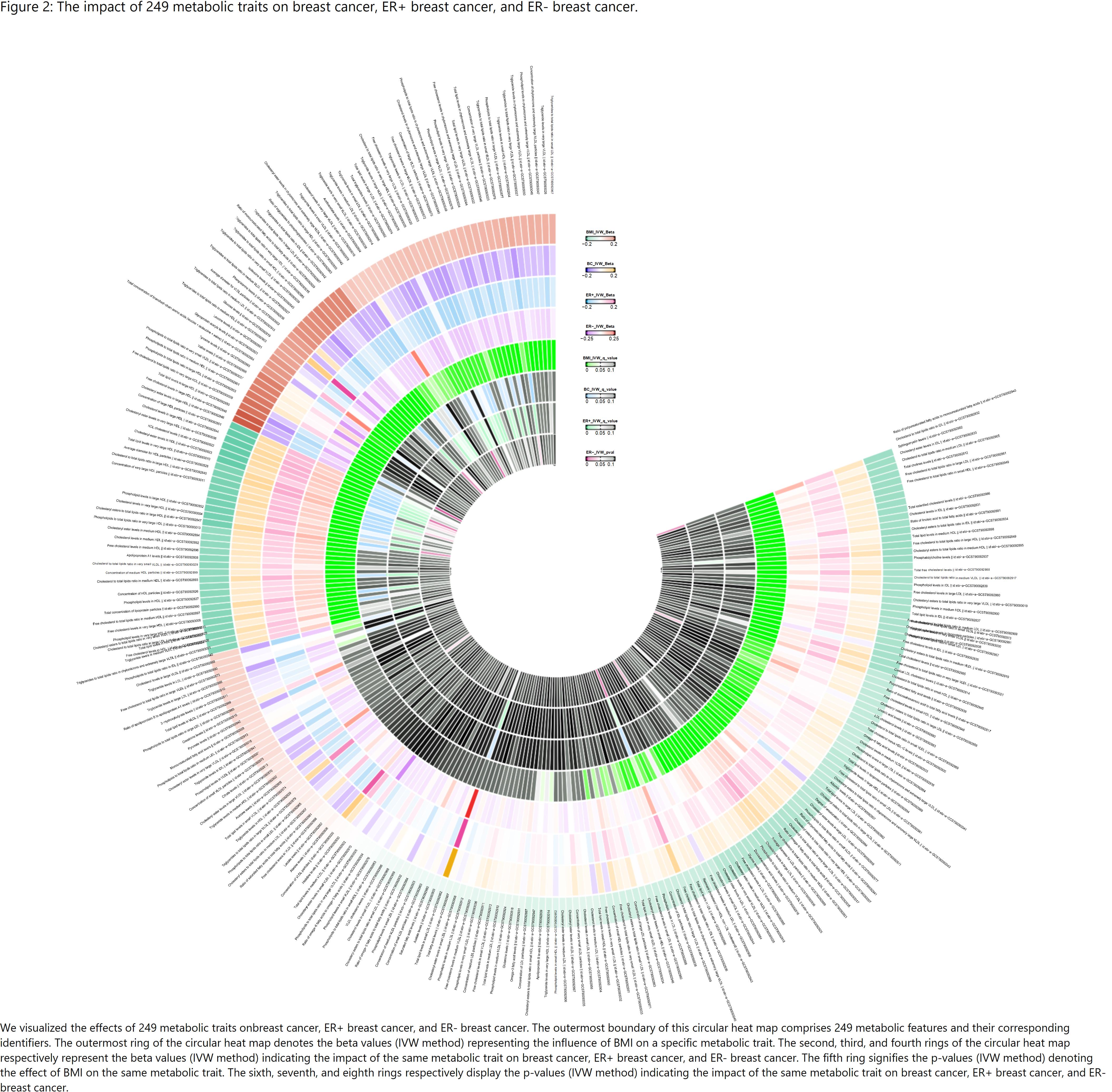

Supplement: Supplementary file 2 [file Image1.jpg]
